# Digitally Optimizing the Information Flows Necessary to Manage Professional Athletes: A Case Study in Rugby Union

**DOI:** 10.3389/fspor.2022.850885

**Published:** 2022-06-09

**Authors:** Jayamini Ranaweera, Dan Weaving, Marco Zanin, Matthew C. Pickard, Gregory Roe

**Affiliations:** ^1^Carnegie Applied Rugby Research (CARR) Centre, Carnegie School of Sport, Leeds Beckett University, Leeds, United Kingdom; ^2^Bath Rugby Football Club, Bath, United Kingdom; ^3^Leeds Rhinos Rugby League Club, Leeds, United Kingdom

**Keywords:** sports informatics, digitization in sport, system usability assessment, sport process optimization, Business Process Management

## Abstract

Practical case studies elaborating end-to-end attempts to improve the quality of information flows associated with athlete management processes are scarce in the current sport literature. Therefore, guided by a Business Process Management (BPM) approach, the current study presents the outcomes from a case study to optimize the quality of strength and conditioning (S&C) information flow in the performance department of a professional rugby union club. Initially, the S&C information flow was redesigned using integral technology, activity elimination and activity automation redesign heuristics. Utilizing the Lean Startup framework, the redesigned information flow was digitally transformed by designing data collection, management and visualization systems. Statistical tests used to assess the usability of the data collection systems against industry benchmarks using the System Usability Scale (SUS) administered to 55 players highlighted that its usability (mean SUS score of 87.6 ± 10.76) was well above average industry benchmarks of similar systems (Grade A from SUS scale). In the data visualization system, 14 minor usability problems were identified from 9 cognitive walkthroughs conducted with the High-Performance Unit (HPU) staff. Pre-post optimization information quality was subjectively assessed by administering a standardized questionnaire to the HPU members. The results indicated positive improvements in all of the information quality dimensions (with major improvements to the accessibility) relating to the S&C information flow. Additionally, the methods utilized in the study would be especially beneficial for sporting environments requiring cost effective and easily adoptable information flow digitization initiatives which need to be implemented by its internal staff members.

## Introduction

**Figure 1:**
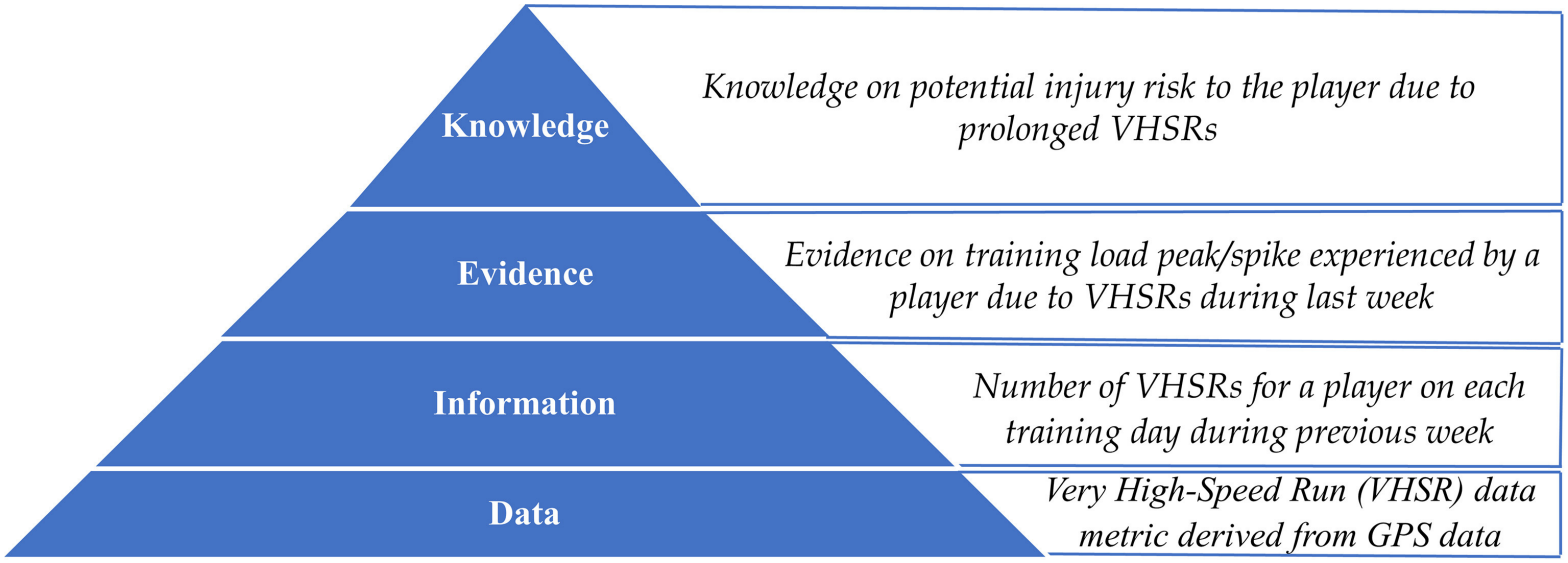
Data-Information-Evidence-Knowledge (DIEK) hierarchy introduced by Dammann (2018) in the Online Journal of Public Health Informatics (OJPHI) with a professional rugby union illustration.

Sport practitioners in the modern era rely heavily on evidence generated from data and information sources to manage professional athletes (West et al., 2019). Based on initial information and knowledge management frameworks such as the *wisdom hierarchy* (Rowley, 2007), researchers like Dammann (2018) have illustrated the process of generating knowledge from data as a four-layer hierarchical model (Figure 1). Experts have defined data, information and knowledge through different viewpoints (Zins, 2007) and it is therefore challenging to provide a single valid definition for data. However, it is assumed that data on its own has no meaning which has led to defining information as *data with meaning* (Hey, 2004). Researchers have further illustrated that information is a flow that leads to the creation of knowledge as a stock (Scharmer, 1996). Therefore, an information flow might initiate from data and extend until knowledge creation. This demonstrates that data and information act as the principal foundation for generating evidence and knowledge. Hence, in sporting environments, where information plays a significant role in generating the evidence and knowledge necessary to manage athletes (refer to Figure 1 for an example from rugby union), it is vital that sport practitioners have access to high quality data and information sources to support decision making. Additionally, a suboptimal information flow at a micro-level can lead practitioners to generate incorrect or incomplete decisions pertaining to player management (e.g., practitioners making incorrect judgments on player training load due to inaccessible information). This in turn, could deviate the player management outcomes from the overall organizational goals (e.g., enhanced injury risks to the athlete or suboptimal training adaptations).

Therefore, minimizing noise and enhancing the quality of information flows linking to player management processes through optimization would enable the creation of rigorous decision support systems in professional sporting environments (Schelling and Robertson, 2020) and enhance the overall effectiveness of decision making associated with player management. Extending this line of thinking further, there has been a growing interest to embed modern strategies such as digital transformation into sporting environments (Ströbel et al., 2021). While information system architectures for sports specific environments have been discussed on the macro-level (Blobel and Lames, 2020), limited practical case studies exist to highlight how such digitization attempts can be implemented by sport practitioners in professional sporting environments at the micro-level (Mullane et al., 2011). Furthermore, in current contexts, implementing data visualization systems to illustrate sport specific information appears to be the favorable method to optimize the information flows in sporting environments (Perin et al., 2018; Du and Yuan, 2021).

However, developing a data visualization system would only act as a part of a complete digital optimization project. For instance, for digitization projects, sport literature does not currently provide sufficient practical case studies to elaborate how requirements for such data visualization systems were generated in sporting environments, how the usability of those systems (e.g., data visualizations) were evaluated and how assessments were conducted to determine if such systems truly enhanced the performance of a given sporting environment (relating to a performance dimension of time, cost, quality or flexibility). Hence, based on digitization techniques, this article aimed to bridge this gap in sport literature by presenting the results from an attempt to improve the quality of an information flow associated with player management processes in a professional rugby union environment. Specifically, based on a practically implemented case study, the current article aimed at; (1) redesigning a player management information flow to overcome an existing issue in a professional sporting environment (2) implementing the redesigned information flow using digitization techniques (3) evaluating the usability of the implemented digital systems (4) assessing the change in information quality due to the optimization.

## Methods

### Case Study

The present study concentrated on optimizing the information flow of player management processes at a rugby union club competing in the Gallagher Premiership in England. During the 2019/2020 season, an organizational objective was set at the club to enhance the use of information for decision making within the performance department, referred to as the High-Performance Unit (HPU). At the time frame of the study, the HPU consisted of physiotherapists (5), strength and conditioning (S&C) coaches (3), sports scientist (1), doctor (1), medical administrator (1) and a data scientist (1). Additionally, Head of Medical, Head of Strength and Conditioning and Head of Applied Sciences and Research were providing leadership to the HPU and will be referred to as the *HPU management team* throughout the article. Furthermore, the study was approved by the ethics committee of the affiliated university.

**Figure 2:**
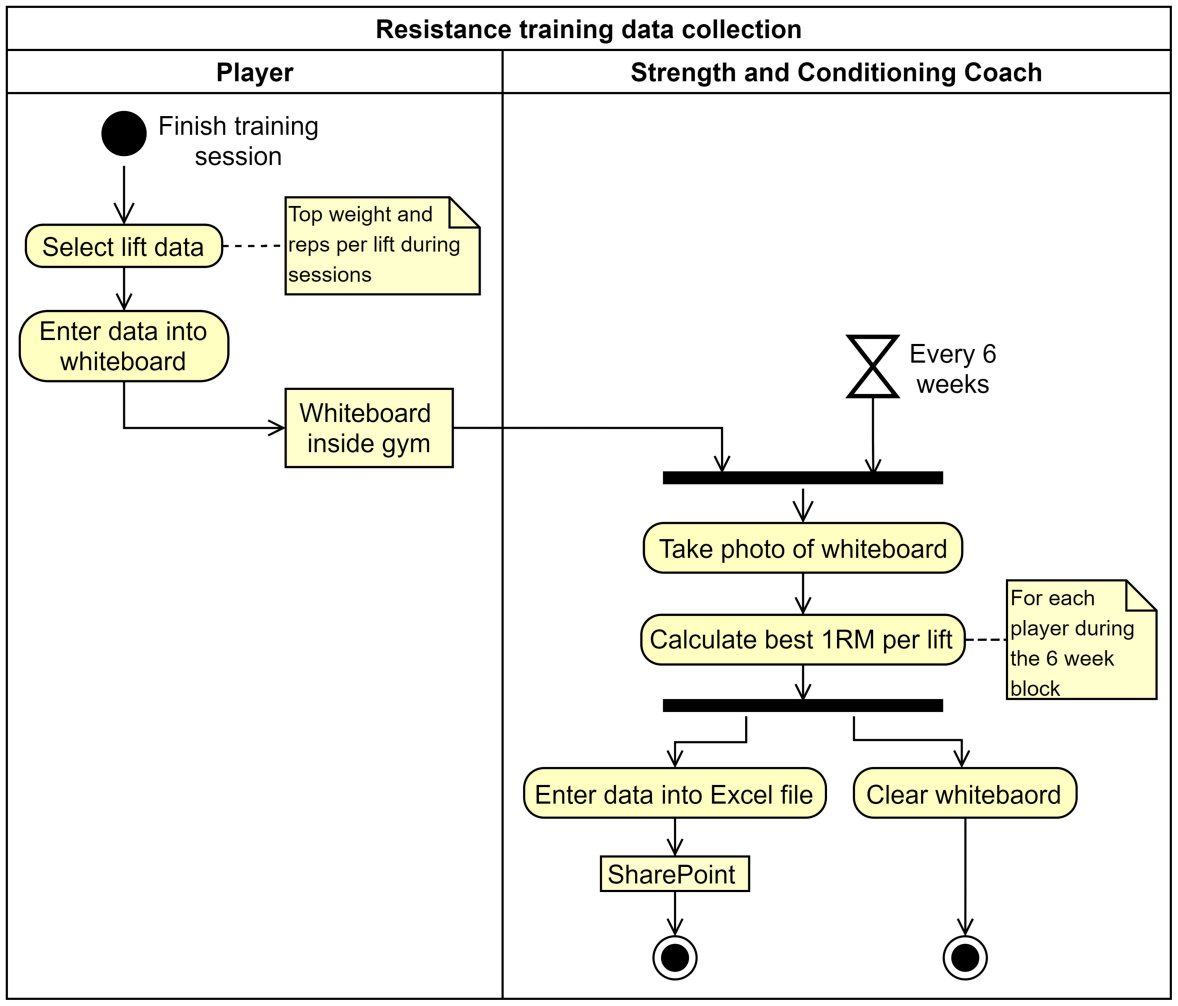
Activity diagram of resistance training data collection prior to the optimisation (will be referred to as As-Is state).

As the first step of this project, specific issues existing in the HPU player management information flow were identified. Among them, the inaccessibility of strength and conditioning data (includes resistance training data for each training day and progressive baseline testing data) of the players was one of the key issues in the current state information flow. For illustration, using an activity diagram in Unified Modeling Language (UML), Figure 2 demonstrates how resistance training data was collected prior to the optimization. The model shows that only one data point for a 6-week period was available online to be accessed by the HPU staff. But if access to daily player resistance training data was required for decision making, then the practitioners could only obtain that data by observing the whiteboard inside the gym. Therefore, the rest of the article will present the outcomes from the attempts that were undertaken to optimize the strength and conditioning information flow (resistance training and baseline testing) of the players within the HPU (results will be mainly provided for improvements relating to the resistance training information flow and where appropriate, progressive baseline testing information flow optimization outcomes will also be presented). During the change management initiative, special attention was given to improve the accessibility of the S&C information flow to HPU staff during decision making.

### Business Process Management (BPM) Approach

**Figure 3:**
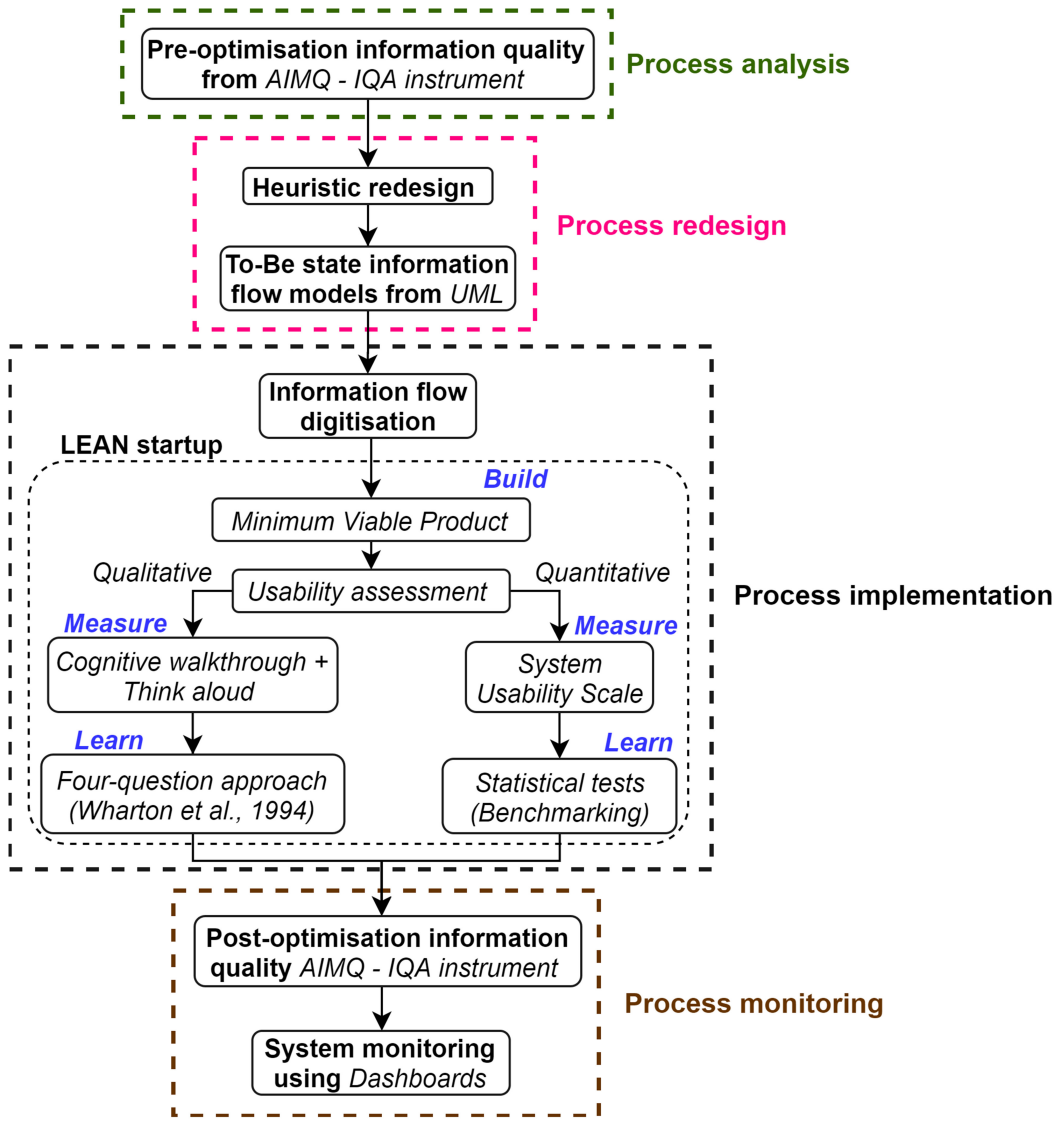
The methods adopted to optimize the strength and conditioning information flow in the HPU.

We have adopted Business Process Management (BPM) as the change management framework to optimize the considered information flow in the HPU. The applicability of BPM to sporting contexts has been discussed previously (Ranaweera et al., 2021) and as specified below, we have adopted the six phase BPM lifecycle presented by Dumas et al. (2013). Within the latter lifecycle, the first three phases (process identification, discovery and analysis) were previously implemented to determine the issues existing in the HPU player management information flow (Ranaweera et al., 2022). Hence, in the current article, as presented in Figure 3, the last three stages (process redesign, implementation, and monitoring) of the BPM lifecycle were used to improve the S&C information flow in the HPU. However, the current study was initiated by assessing the quality of the considered information flow prior to optimization. This latter step is a constituent of the process analysis stage.

- Process identification—Generates the organizational process architecture, performance measures, relationships and systematically identifies which of those processes require a BPM intervention to assist in meeting organizational goals.
- Process discovery—For the identified processes, information on the current state (As-Is) is collected and documented through process modeling techniques.
- Process analysis—Issues within the documented As-Is state of the processes are identified for optimization, potentially generating a list of prevailing issues.
- Process redesign—The process is redesigned (optimized) to overcome the issues identified in the process analysis stage to define the best future state (To-Be).
- Process implementation—Necessary changes to move the process from the As-Is to the To-Be state are performed by managing organizational change.
- Process monitoring—The implemented process is monitored to determine the effectiveness of the changes. The cycle is repeated to discovery stage if further issues are present or further continuous improvements are necessary.

### Process Analysis

#### Pre-optimization Information Quality Assessment

Research has discussed data quality as an outcome from decision support systems proposed for high-performance sporting environments by considering the effects to different dimensions (e.g., reliability, accessibility, timeliness) of data quality (Schelling and Robertson, 2020). Wider computing research illustrates different frameworks to assess data quality (Batini et al., 2009; Cichy and Rass, 2019) including combined subjective and objective methods (Pipino et al., 2003). However, we opted to refer to the assessment of information quality rather than data quality as theoretically (from the DIEK hierarchy in Figure 1) practitioners are more inclined to create evidence and knowledge from information sources rather than direct data sources (Dammann, 2018).

Hence, we used the product and service performance model for information quality (*PSP/IQ model)* (Kahn et al., 2002) to assess the quality of the S&C information flow in the HPU. This focuses on assessing the information quality from 16 dimensions that are mapped to four quadrants: sound, useful, dependable and usable information. Within the PSP/IQ framework, the view of information as a product (data generation to storage, typically in a database) or service (transforming data to information) is closely related to the DIEK hierarchy and its illustration in sport. For example, the collection, storage and maintenance of daily S&C data can be viewed from *information as a product* lens. The process of accessing the stored S&C data and visualizing it as an information source to staff for player management can be analyzed from *information as a service* viewpoint. We utilized the standard *IQA instrument* (questionnaire with 11-point scale), introduced through the *AIMQ* information quality assessment method (Lee et al., 2002) (built on the foundations of the PSP/IQ model) to subjectively determine the information quality of the information flow considered for optimization. Therefore, at the initiation of the study, the IQA questionnaire was administered to all the members of the HPU (refer to Table 1 for the participant characteristics) who required access to daily S&C data of the players in the first team and senior academy. The feedback to the questionnaires were used to determine the pre-optimization information quality level of the S&C information flow.

**Table 1:** Information quality assessment and cognitive walkthrough participant characteristics.

**User** **ID**	**Age**	**Years of experience in professional sport**	**Pre-optimization**	**Post-optimization**	**Cognitive walkthrough**
U_1_	35	8	Y	Y	Y
U_2_	39	14	Y	Y	Y
U_3_	37	11	Y	Y	Y
U_4_	27	5	Y	Y	Y
U_5_	31	6	Y	Y	Y
U_6_	46	12	Y	Y	Y
U_7_	29	8	N	Y	Y
U_8_	31	4	N	Y	Y
U_9_	27	4	N	N	Y

### Process Redesign

For the current study, we used *Heuristic Redesign* (Reijers and Mansar, 2005; Dumas et al., 2013) to transform the S&C information flow into an improved future state. Heuristic redesign was suitable for redesigning player management processes since it focuses on changing the current (As-Is) state incrementally (transactional) whilst operating within the context of the As-Is process (inward-looking) by using a defined set of redesign heuristics (analytical). Readers can refer to the redesign orbit presented by Dumas et al. (2013) to understand other process redesign techniques. Furthermore, most outward-looking redesign strategies (e.g., redesigning from best practices on similar processes by other alike sporting organizations) were not practical as sport organizations tend to operate within closed boundaries and do not often consider strategies such as open innovation. On a specific level, we selected relevant redesign heuristics from the 29 presented by Dumas et al. (2013) to transform the considered information flow to an optimized future state (To-Be). The resulting processes after redesign and the systems necessary to transform the processes were modeled using UML.

### Process Implementation

Once the S&C information flow was redesigned, it was implemented using digitization techniques. Within the literature, the common mode of process implementation in a BPM lifecycle is to automate tasks by using a Business Process Management System (BPMS) (De Ramón Fernández et al., 2019). Whilst there might be possibilities to integrate a BPMS with an Athlete Management System (AMS) in sporting contexts, for the current study, instead of experimenting with a BPMS, we implemented the redesigned information flow by developing digital systems to collect, manage and visualize daily resistance training and progressive baseline testing data of the players. This was because firstly, the implemented system automation had to address unique user requirements aligning to the business goals of the considered organization. Secondly and most importantly, unlike certain other professional sports (e.g., football), sporting organizations like the one considered in the current study did not possess the financial strength that was needed to automate the redesigned process using an existing BPM technology like a BPMS.

**Figure 4:**
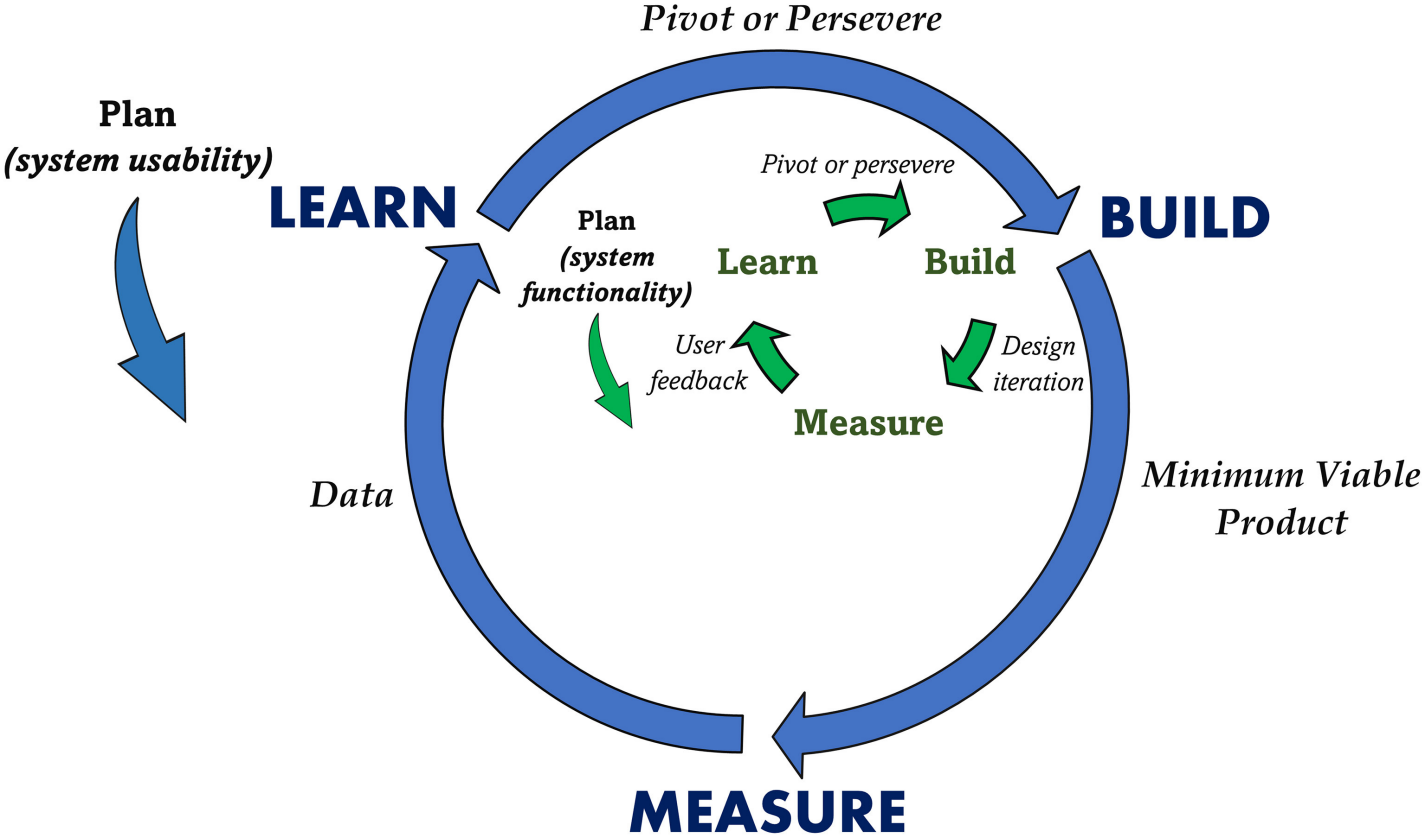
Build-Measure-Learn cycle in Lean Startup framework (Reis, 2011).

Due to the dynamic nature of the HPU (e.g., constantly changing requirements), system developments needed to be flexible and agile in nature. Therefore, as highlighted by other sport researchers (Lacome, 2020), a method like *Lean Startup* (Reis, 2011), which focuses on a more adaptable process to changes, consisting of shorter design durations with early prototyping during the product development lifecycle, was more suitable for developing the proposed data collection, management and visualization systems. Specifically, guided by a design thinking approach for collecting the user requirements (Blessing and Chakrabarti, 2009), as illustrated in Figure 4, we utilized the iterative *build-measure-learn* cycle proposed in Lean Startup to digitize the S&C information flow.

#### Plan

Although the build-measure-learn cycle appears to initiate with the build stage, it practically begins in the plan step, occurring in reverse to the development flow. Basically, a hypothesis about the system (e.g., if staff use the system for certain number of times, then it can be deemed appropriate to be implemented) is defined first through the plan step. Subsequently, the resulting build-measure-learn cycle is executed to understand if the hypothesis set in the plan step is proven, if not, the cycle is repeated. For the current study, as illustrated in Figure 4, we used two build-measure-learn loops for the system development. An internal loop (executed entirely within the Build phase of the outer loop) was used to design a *Minimum Viable Product* (MVP) to meet the functionality requirement and the outer loop was used to validate its usability.

For the internal loop, we defined the exact user requirements (functional) from the proposed data collection, management and visualization systems in the plan stage. At the onset of the system development, we embedded three users into the design cycle to guide the design process. Specifically, two S&C coaches (each from the first and academy teams) and one physiotherapist were selected for this purpose. Therefore, the initial functional requirements from the systems were collected from unstructured interviews with those three HPU members. The outer loop was targeted at assessing the system usability. For such usability assessments, the following criteria were defined in the plan stage.

- Data collection systems were acceptable if the usability of them were above the industry defined average system usability thresholds for such similar systems.
- Data visualization systems were acceptable if no major usability problems were existing in them during operation.

#### Build

The principal outcome from the build phase was to create a Minimum Viable Product (MVP) (Lacome, 2020), which was used to validate the usability criteria defined in the plan stage. An MVP is a version of the system providing just enough features to be usable by the customers for evaluating the prior set standard in the plan stage. Readers can refer to the original work by Reis (2011) to understand the different available options for developing MVPs. For the current study, we followed the internal build-measure-learn loop to design an MVP to meet the basic functional requirements set by the users. Specifically, for the S&C information flow, we first developed web based interfaces coupled with Internet of Things (IoT) (Ikram et al., 2015) based gateways to collect daily player data and then implemented a Database Management System (DBMS) (Vincent et al., 2009) to manage it. We designed web-based interfaces to process and visualize the data as meaningful information to staff. Therefore, each build iteration in the internal loop produced a design version of the MVP. Next, we collected feedback (functionality) regarding each design version from the three users in the design loop (measure). Finally, the comments were validated against the functionality requirements (learn) to understand if another design iteration within the internal loop was necessary or if the development could progress to the next phase (measure) of the outer build-measure-learn loop. Once the system functionality requirements were met through the internal build-measure-learn loop, that final version of the system was defined as an MVP of the outer loop.

#### Measure

After building an adequately functioning MVP, measurements relating to the usability of the MVP were necessary to evaluate it against the criteria set in the plan stage (system usability requirements). Therefore, this step was associated with collecting data about the usability of the MVP. There were two distinctive MVP systems relating to the digitized resistance training information flow; (1) data collection systems (2) data visualization system. Therefore, aligning to the plan phase, as specified below, two different approaches were undertaken to collect data on the usability of the two systems.

- *Data collection system:* In relation to the plan step, the goal was to assess the usability of the system in reference to industry benchmarks (summative). Hence, for this purpose, we chose the System Usability Scale (SUS) (Brooke, 1995) from the different standard usability assessment questionnaires defined in literature (Sauro and Lewis, 2016) to collect data on the usability of the system. Therefore, the goal was to collect the necessary SUS scores for the interface to be assessed against the industry benchmarks of similar interfaces defined in literature (Bangor et al., 2008). To achieve this, first, a priori sample size calculation [α = 0.01, 80% power, *SD* = 8.54 from a similar study (Yuliawan et al., 2020)] was conducted to determine the minimum number of individuals that were required to obtain a statistically significant assessment on the usability of the system to be at least 1 score above the SUS average industry benchmark score of 68 (Sauro and Lewis, 2016). This resulted in a sample size of 852 (Supplementary Data Sheet 1), which is not practically viable in any sporting environment. In the considered rugby club, the squad at the time frame had only 65 players. Therefore, after the players had used the data collection interface to enter their daily resistance training data following a session, author JR administered the SUS questionnaire to 55 players (*mean age* = 24.11±4.26) in the squad (all the available players) and requested them to rate the usability of the system using the scale. The unavailable athletes for data collection (*n* = 10) were either injured or on loan at another club.
- *Data visualization system:* According to the criteria set in the Plan phase, the goal was to collect data to unravel potential usability issues in the system (formative). Hence, we conducted cognitive walkthroughs (Mahatody et al., 2010) with think aloud techniques (users discussing their thoughts when interacting with a system during testing) (Nielsen, 2012; Alomari et al., 2020) with 9 HPU staff members (refer to Table 1 for the participant characteristics) to identify usability problems in the data visualization interface. To achieve this, in the HPU, we set up a lab-based environment where a participant would attempt a set of tasks using the interface under the presence of a moderator (Supplementary Figure 1). Specifically, the five tasks (covering each core aspect of the interface) mentioned in Table 2 were developed for the cognitive walkthrough sessions. Author JR conducted those sessions (each <30 min) by acting as the moderator. During the sessions, users were requested to attempt those tasks (order of tasks were randomized for each user) using the data visualization interface and they recorded the results from each task on an online task sheet. Additionally, after completing each task, users were requested to answer the Single Ease Question (SEQ) (Sauro and Lewis, 2016; Laubheimer, 2018) to help determine the difficulty of the task. And screen recording was used to collect audio (users think aloud data) and video (screen) data when the users attempted the tasks.

**Table 2:** Description of tasks designed for the cognitive walkthrough sessions.

**Task**	**Task description**
T_1_	Record the All-Time best Squat one-repetition maximum (1RM) value of player A.
T_2_	Record Current (42 day rolling) best Bench Press 1RM rating of player B.
T_3_	State the recorded Dumbbell Press weight, reps and lift type by player C on 3/3/2021
T_4_	Determine (Yes/No) if player D had done his Calf ISO's during week number 7 of year 2021.
T_5_	For player D, during the period of 1/12/2020 to 31/03/2021, please determine the lowest SL CMJ Jump Height Left, Right values and record if it is Good/Poor.

#### Learn

The final phase of the cycle was used to analyze the collected data from the measure step to determine if the MVP met the usability criteria set in the plan step. In line with the measure step, two distinctive approaches were considered for the two systems.

- *Data collection system:* To validate the usability, first, a random sample of 50 SUS scores were selected from the data collected from all the active players. Next, as per the arguments justifying the use of parametric tests to group ordinal data like SUS scores (Harpe, 2015; Sauro and Lewis, 2016), a one-sample *Z*-test (by setting α = 0.01) was used to determine if the mean SUS scores of the sample (x) were statistically different (above) from the average industry SUS score of 68 (μ) (Bangor et al., 2008; Sauro and Lewis, 2016). Additionally, the mean SUS score was also compared against a SUS grading scale defined in literature (Sauro and Lewis, 2016) to determine an overall grading (comparative) for the usability of the data collection interface in relation to similar interfaces defined in literature. Finally, if the usability of the system was above industry average, it was decided to *Persevere* with the system by implementing any minor improvement. On the contrary, if the usability was below average, the cycle was repeated by using another internal build-measure-learn loop, a new MVP was created by *Pivoting* to the build step of the outer loop.
- *Data visualization system:* For identifying any usability issues in the system using the cognitive walkthroughs, first, the action sequences of each task (series of steps required to successfully execute the given task) were defined. Next, as specified below, author JR used the four-question approach by Wharton et al. (1994) to walk through the action sequences of each task performed by all the users (using the screen recordings). In this context, for each action in the task, a Yes/No was marked against the four questions and a rating of No for any question illustrated a usability problem in that action.
  ○ Will the user try to conduct the right action (Q1)?
  ○ Will the user recognize that the correct action is available (Q2)?
  ○ Will the user know that the correct action will achieve the expected outcome (Q3)?
  ○ If the correct action is performed, will the user notice that progress is being made toward achieving the final outcome of the task (Q4)?

Finally, the validity and severity (minor vs. major) of the unraveled usability problems were evaluated with the authors DW and GR. In relation to the plan step, if no major usability problems were present, it was decided to *Persevere* with the existing design by conducting any minor amendments. Otherwise, a new MVP was developed by *Pivoting* to the build step and the build-measure-learn loop were repeated.

### Process Monitoring

#### Post-optimization Information Quality Assessment

Once the S&C information flow was digitally optimized, the same IQA instrument in the AIMQ information quality assessment method was administered to the HPU staff to determine the post-optimization information quality level. Table 1 illustrates the characteristics of the HPU practitioners (pre-post) who responded to the questionnaire. The resultant pre-post optimization information quality change for all the dimensions and the corresponding impact to each quadrant of the PSP/IQ model were analyzed. However, due to the low sample sizes, statistical tests were not conducted to statistically determine the pre-post impact to the information quality of the considered information flows.

## Results

As specified previously, for illustration, the main results relating to the S&C information flow optimization will be presented. Specifically, results will be mainly provided for improvements relating to the resistance training information flow and where appropriate, progressive baseline testing data optimizations outcomes will also be presented.

### Process Redesign

As specified below, three key redesign heuristics were used to transform the S&C information flow to an optimized future state (To-Be).

**Figure 5:**
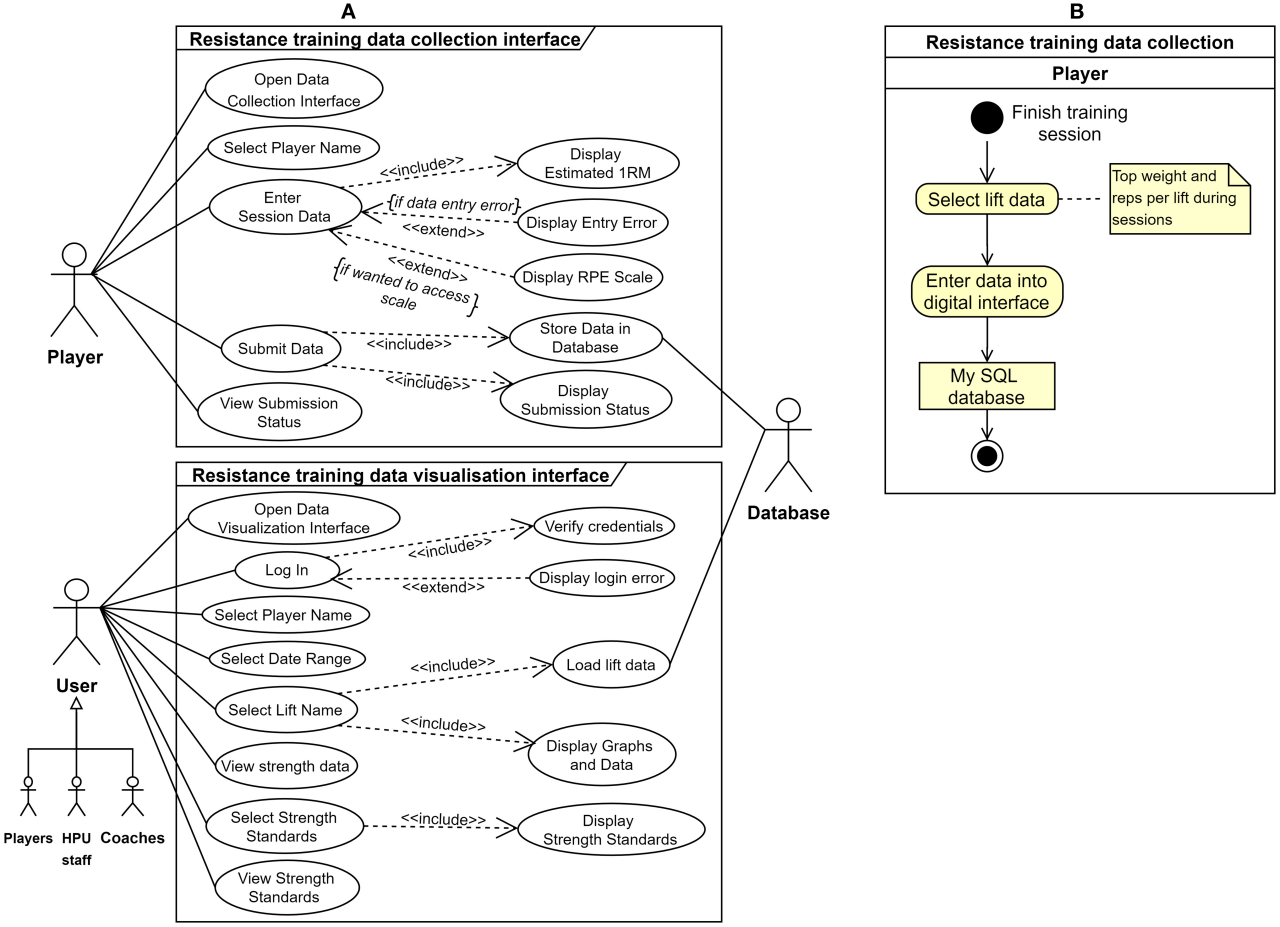
Redesigned resistance training information flow **(A)** use case diagram from UML depicting the proposed system **(B)** resistance training data collection future state (To-Be) process model. Notice how all the activities of the S&C Coach have been eliminated (refer to Figure 2 for As-Is state).

- Integral technology (to introduce new technology for process execution): For the resistance training information flow, instead of recording data on a whiteboard, as presented by the *use case diagram* in Figure 5A, a new digital interface was proposed to allow the players to enter their daily resistance training data after each session, and the collected data was planned to be stored and managed within a database. A similar interface was also proposed to enter the progressive baseline testing data of the players. However, data entry to the latter system was to be done by HPU staff and not the players. Next, a data visualization interface would access the daily S&C data in the database and visualize the necessary information to the HPU staff.
- Activity elimination (to remove unwanted activities from the process): All the activities performed by the S&C coach in the previous resistance training data collection process (Figure 2) would be eliminated to create an optimized future state (Figure 5B).
- Activity automation (considers automation of tasks in the considered process): The proposed data visualization interface automated tasks (e.g., estimated 1RM calculation, graphical representation of data) currently executed in the As-Is state when accessing S&C information by HPU staff.

### Process Implementation

For illustration, the results from the attempts to implement the high-level system described in Figure 5 for optimizing the resistance training information flow will be presented. In such regard, as per the methods, the digital system was implemented using the two (internal and external) build-measure-learn loops.

#### Plan

For the Plan step, Table 3 presents the functionality requirements of the resistance training data collection system, discovered from the unstructured interviews with three users connected to the design cycle. Similar requirements were defined for the baseline testing data collection and the overall data visualization interface as well (Supplementary Table 1).

**Table 3:** User defined functionality requirements of the resistance training data collection system.

**Requirement**	**Detail**
Data entry features	The data entry user must be selected by the player.
	For each of the defined lifts, users must be able to enter the weight and repetitions corresponding to their top lift for the session.
	Users must be able to select the lift type (e.g., heavy or dynamic) for lower body lifts.
	Classes of lift types (e.g., upper body, lower body, prophylactics) must be grouped.
	Separate data entry inputs are necessary for player body weight and Rated Perceived Exertion (RPE).
	Prophylactic data requires a 'Yes/No' input.
Real-time feedback	Automatically calculate and show the 1RM value corresponding to the entered weight and reps to the player.
	Notify users of the data entry status, including any specific data entry errors.
	Notify users the success/failure of data submission.
Data entry controls	Restrict users from submitting incorrect data. The interface must be capable of detecting defined data entry error conditions.
	Restrict users from submitting data if mandatory data inputs are not entered (e.g., player body weight data is mandatory to calculate relative strength scores).
	Users must enter data to at least a single lift to submit the data for storage.
	Prevent players from entering multiple data entries for a specific date.
Data storage	The entered data must be stored in a secure database.
User friendliness	The interface must be simple and easy to use. The number of operations performed by the user must be minimized.
Accessibility	The interface must be easily accessible to the players after completing their resistance training sessions.
	The RPE scale must be accessible to the player during data entry.

#### Build (External Loop)

**Figure 6:**
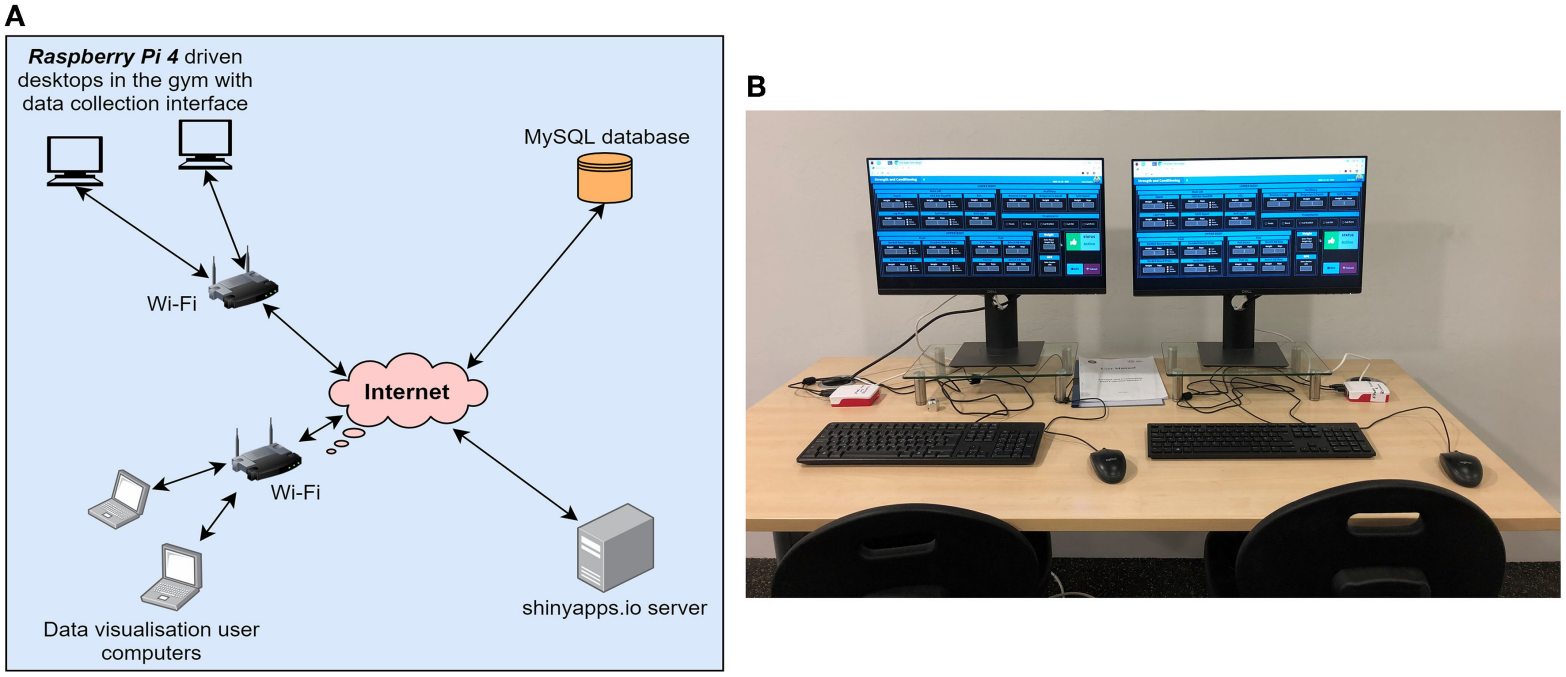
Implemented system overview **(A)** network model **(B)** data collection interfaces inside the gym.

From a technical point of view, to implement the redesigned resistance training information flow, as shown by the system network model in Figure 6A, we developed a web-based interface using R Shiny (application hosted in shinyapps.io server) for collecting the daily resistance training data of the players. The data collection interface was launched inside the gym using Raspberry Pi 4 devices (Figure 6B), acting as IoT gateways using Wi-Fi and the collected data were stored in a MySQL database. Finally, data visualization interfaces were also designed using R Shiny (hosted in shinyapps.io server) to allow HPU staff, players and coaches to automatically access the data in the database when necessary and transform it to meaningful information to support decision making relating to player management.

**Figure 7:**
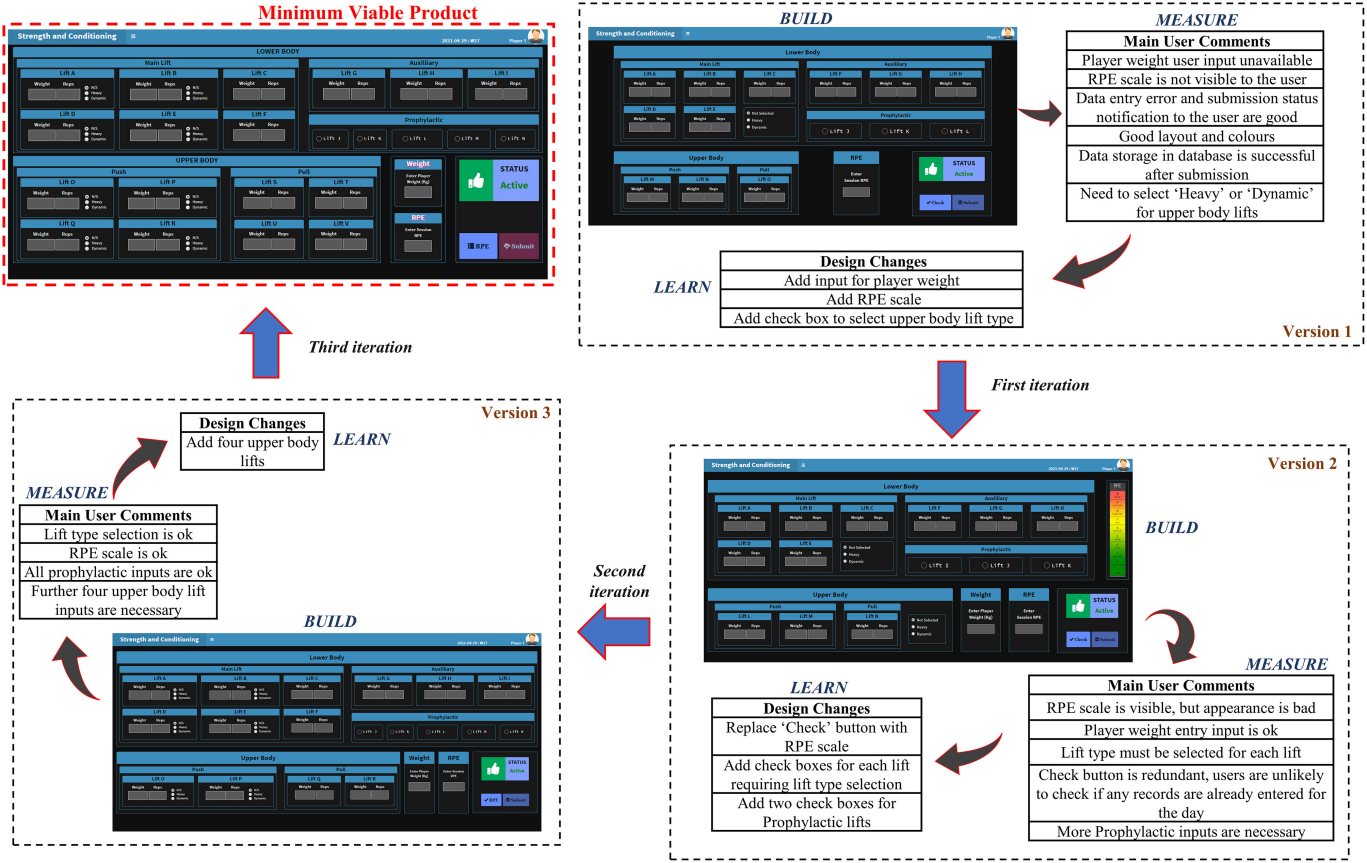
Internal build-measure-learn loop used to develop the digital interface for daily resistance training data collection MVP.

**Figure 8:**
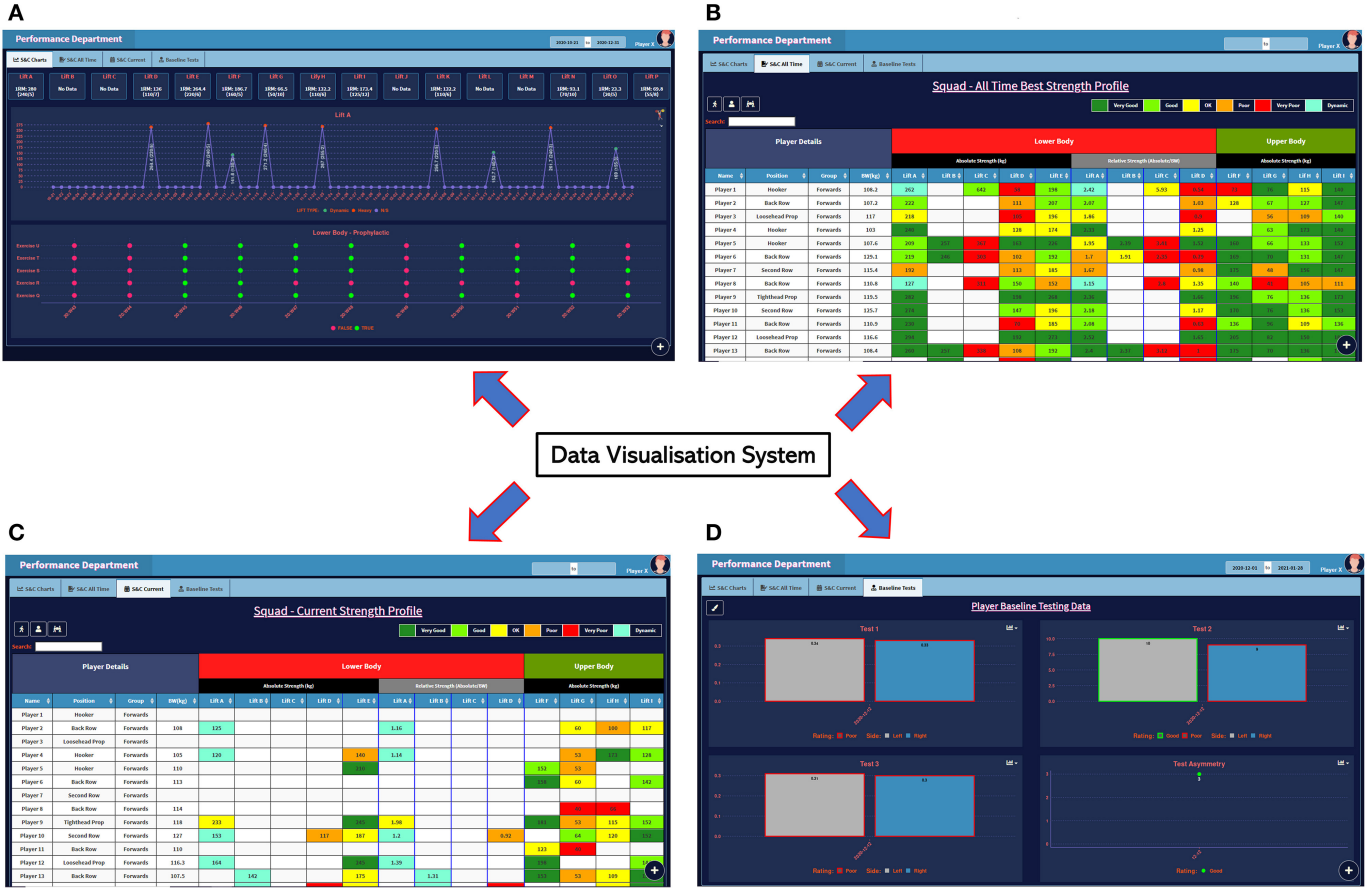
Strength and conditioning data visualization system (interface) MVP **(A)** S&C Charts **(B)** S&C All Time **(C)** S&C Current **(D)** Baseline Tests.

Utilizing the technologies discussed previously, the internal build-measure-learn loop within the Build step of the external loop was used to develop an MVP to achieve the functionality requirements defined in the Plan step (e.g., the requirements for the data collection interface defined in Table 4). Figure 7 illustrates each iteration of the internal build-measure-learn loop used to design the resistance training data collection system MVP, including each version, the corresponding feedback by the three users in the design loop and the required design changes for the next iteration. Readers can refer to Supplementary Figure 2 for the algorithm flow of the data collection system. Figure 8 shows the data visualization system MVP and specifically, as described below, the four tabs illustrate how S&C data were converted to the different information sources which were necessary to manage the athletes.

(A) S&C Charts (for each player)
  - Illustrates the best scores, per each resistance training exercise, during a selected date range.
  - Presents the longitudinal data (each day of the selected date range) of each exercise.
  - Based on a color coding, indicates (Yes/No) if each prophylactic exercise has been conducted at least once during a week.
(B) S&C All Time (whole squad)
  - A table illustrating the best scores of all players in the squad for each resistance training exercise (calculated from all data in the database until the current date).
  - Within the performance department, according to the positional groups in rugby union (e.g., prop, scrum half), the expected standards for each resistance training exercise have been defined. Therefore, aligning to the methods defined in sport literature (Robertson et al., 2016), each data cell in the table was color coded in relation to the set standards.
(C) S&C Current (whole squad)
  - A table illustrating the best scores of all players in the squad for each resistance training exercise performed during the last 6 weeks (rolling). Notice how the missing data (in all columns) for players illustrate that they were either injured, on loan or training with the national team.
  - The same color coding described previously was used to highlight each cell.
(D) Baseline Tests (for each player)
  - Illustrates the best scores, per each baseline test, during a selected date range.
  - At a given instance, four different tests can be compared on the screen.
  - Similar to the above, standards have been defined for each baseline test and they were incorporated into the graphs by translating into a color coding.

**Table 4:** Task completion rates (including SEQ results) for the usability evaluation data collection.

**Task** **number**	**User**																		**Task completion rate *(%)***	**SEQ (Geometric mean)**
	**U_1_**		**U_2_**		**U_3_**		**U_4_**		**U_5_**		**U_6_**		**U_7_**		**U_8_**		**U_9_**			
	**Completion**	**SEQ**	**Completion**	**SEQ**	**Completion**	**SEQ**	**Completion**	**SEQ**	**Completion**	**SEQ**	**Completion**	**SEQ**	**Completion**	**SEQ**	**Completion**	**SEQ**	**Completion**	**SEQ**		
T_1_	Y	7	Y	7	Y	5	Y	7	Y	7	Y	7	Y	6	Y	6	Y	5	100	6.46
T_2_	Y	7	N	7	N	5	Y	7	N	7	N	1	N	6	N	6	Y	5	33.33	5.06
T_3_	Y	7	N	7	N	3	Y	6	Y	6	Y	5	N	6	N	3	Y	4	55.56	5.13
T_4_	Y	7	Y	5	Y	5	Y	5	Y	7	N	5	Y	6	Y	4	N	6	77.78	5.41
T_5_	Y	7	Y	4	Y	2	Y	5	Y	7	Y	3	Y	6	Y	4	Y	6	100	4.40

#### Measure (External Loop)

The data collected to evaluate the usability of the two MVP's described previously; (1) data collection (Figure 7) and (2) data visualization (Figure 8) is specified below.

*Data collection system:* The mean (x) System Usability Scale (SUS) score of the random sample (*n* = 50) was 87.6 (*SD* = 10.76), where the 99% confidence interval (CI) ranged from 83.68 to 91.59 (Supplementary Data Sheet 1).

*Data visualization system:* For data collection relating to the cognitive walkthrough (usability evaluation of the data visualization system), the completion rates of the 5 tasks performed by the HPU staff and the responses to the Single Ease Question (SEQ) question are specified in Table 4. Although users had found Task 5 to be the most difficult, everyone had successfully competed it along with Task 1. On the contrary, Task 2 had the worst completion rate among the five tasks, illustrating the possibility for usability problems associated with it. Additionally, in reference to the geometric mean of the SEQ rating, the users had encountered Task 1 to be the easiest to complete.

#### Learn (External Loop)

From the data collected in the previous step, in relation to the resistance training information flow, the following results were generated relating to the usability assessment and evaluation of the two systems (data collection and visualization).

- *Data collection system:* Initially, aligning to the guidelines suggested for using parametric tests for grouped ordinal data like the Likert-scale in SUS (Harpe, 2015), a computation using R was conducted to evaluate if the central limit theorem (CLT) held true for the sampling considered in the current study. The results demonstrated that the sample means were normally distributed (refer to Supplementary Data Sheet 1). Therefore, the planned Z-tests were conducted for the random sample specified in the Measure step. The core outcomes of the two-sided Z-tests have been provided below; readers can refer to Supplementary Data Sheet 1 in the supporting information for all the calculations.
  ○ An initial one-sample *Z*-test was conducted to evaluate the sample SUS score mean (x = 87.6) against the average SUS industry benchmark (μ = 68), with the significance level set to α = 0.01. The resultant test yielded a two-tailed *p value* = 5.93 *x* 10^−38^. Therefore, sufficient statistical evidence was available to reject the null hypothesis (x = 68).
  ○ Next, a second, one-sample Z-test was conducted to evaluate the sample mean (x = 87.6) against the lower industry SUS score benchmark for a Grade A interface (μ = 80.8) according to the SUS grading scale (Sauro, 2011; Sauro and Lewis, 2016). To compensate for the two tests, a *Bonferroni correction* was used by setting α = 0.005. The resultant test generated a two-tailed *p value* = 7.8897 *x* 10^−6^. Hence, it was possible to reject the null hypothesis x = 80.8) for this test as well.
  ○ Finally, a post study power calculation was conducted in relation to the second Z-test performed against the lower Grade A SUS score (x = 87.6, *μ* = 80.8, *α* = 0.05, *n* = 50, *d* = 0.632). The results illustrated that the test had 95% power.

Therefore, based on the prior results from the statistical tests, there was less than 1% chance that a mean SUS score of 87.6 ± 10.76 for a sample of size 50 would be generated from a population (professional rugby union players using the interface in the considered club) with a mean score equal to 68 or 80.8. With additional justification from the sample 99% CI of 83.68 to 91.59, there was sufficient evidence to suggest that the usability of the data collection system was potentially above industry average and could be minimally rated as a Grade A system according to the SUS grading scale. This meant that the latter system had higher perceived usability than 90% of the systems tested within the SUS grading scale (Sauro, 2011; Lewis, 2018). Therefore, it was decided to *Persevere* with this version of the resistance training data collection system MVP by conducting any further minor modifications.

- *Data visualization system:* According to the plan step, the data visualization system was acceptable if there were no major usability issues within the system. Therefore, for illustration, the results from the cognitive walkthrough evaluations of three users conducted from the four-question approach (Wharton et al., 1994) for the action sequence relating to Task 2 has been provided in Table 5. Similar analysis was conducted for all users and tasks in the assessment.

**Table 5:** Four-question cognitive walkthrough corresponding to the second task (T_2_) used to evaluate the usability of the data visualization system.

**Action sequence**			**User**											
			**U_1_**				**U_2_**				**U_3_**			
**No**.	**Action**	**Type**	**Q1**	**Q2**	**Q3**	**Q4**	**Q1**	**Q2**	**Q3**	**Q4**	**Q1**	**Q2**	**Q3**	**Q4**
1	Click “S&C Current” tab	User	N	N	N	N	Y	Y	Y	Y	N	N	N	N
2	Data appears	System	Y	Y	Y	N	Y	Y	Y	N	Y	Y	Y	N
3	Type player name in “Search”	User	Y	Y	Y	Y	Y	Y	Y	Y	NA	NA	NA	NA
4	Filtered data appears	System	Y	Y	Y	Y	Y	Y	Y	N	NA	NA	NA	NA
5	Scroll down to player name	User	NA	NA	NA	NA	NA	NA	NA	NA	Y	Y	Y	Y
6	Read Bench Press fill color	User	Y	Y	Y	Y	N	N	N	N	N	N	N	N
7	Read the color rating from the legend	User	Y	Y	Y	Y	N	N	N	N	N	N	N	N

*A “No” answer to any question depicted an instance of a potential usability problem*.

From the usability evaluation, 14 usability issues and 4 further functionality improvement requirements (which were not captured in the initial user requirements) were unraveled (Supplementary Table 2). Consensus was reached between the three authors on the severity of the usability problems. Specifically, based on a practical viewpoint, it was agreed that the 14 usability issues were minor problems. Among the functionality improvements, two required major changes to be implemented in the system. However, after a discussion with the HPU management team, it was agreed that the two functionality improvements requiring major changes were of low priority. Therefore, it was decided to *Persevere* with this version of the S&C data visualization system MVP by improving the system to overcome the minor usability problems.

### Process Monitoring

#### Pre-post Optimization Information Quality

The main results pertaining to this stage focused on assessing the change in information quality of the S&C information flow due to the optimization. Table 6 shows the responses provided by each HPU staff member to the IQA questionnaire to assess the pre-post information quality of the S&C information flow.

**Table 6:** Individual responses to the IQA questionnaire and the resulting overall scores for the pre- post-optimization information quality assessment of the S&C information flow.

**Information quality dimension**	**Individual scores**																**Overall score**	
	**U_1_**		**U_2_**		**U_3_**		**U_4_**		**U_5_**		**U_6_**		**U_7_**		**U_8_**		
	**Pre**	**Post**	**Pre**	**Post**	**Pre**	**Post**	**Pre**	**Post**	**Pre**	**Post**	**Pre**	**Post**	**Pre**	**Post**	**Pre**	**Post**	**Pre**	**Post**
Accessibility	3	10	1	9.5	5.5	7.8	6	8.8	6	10	4.3	8.5		7.5		9.3	4.3	8.9
													Data unavailable		Data unavailable			
Appropriate amount	4.8	5	0.5	5.5	6	7.5	5	5	4.8	7.5	5.3	5		3.8		2.5	4.4	5.2
Believability	5.8	7	3.5	7.5	6.3	5.8	6	7	7.5	6.8	5	6.5		6.3		5	5.7	6.5
Completeness	3.7	6.8	5	7.2	5.5	6.3	6.2	7.2	4.3	8.3	4.2	7.2		5.7		6.8	4.8	6.9
Concise representation	5	10	3.3	10	6.3	6.5	3	8.3	5	10	4.5	9		9		7.3	4.5	8.8
Consistent Representation	6.3	7	4.3	7.5	4.3	5.8	6.5	6.5	5	7.5	5	7		7		7.5	5.2	7.0
Ease of operation	4.2	5.6	5	6	5.4	4.6	4.2	5.4	5	6	5.4	5.4		5		5.2	4.9	5.4
Free of error	6.3	6.8	5	7.5	6	6.3	6	6.8	3.3	6.8	4.3	6.8		6.5		6.3	5.1	6.7
Interpretability	5.3	5.8	5	6.6	5.5	5.8	5.8	6	4.8	6	4	5.6		5.6		6.8	5.0	6.0
Objectivity	8	8.5	9	10	8.3	8.3	4.8	7.3	6	7.8	4.8	9		8		8	6.8	8.3
Relevancy	7	10	9	10	8.5	8	9	10	9	10	7	9		8		10	8.3	9.4
Reputation	5.8	6	6.5	6	6	5.8	6	7	5	5.8	5.5	7.3		6.5		6.8	5.8	6.4
Security	5.8	7.5	5.8	7.5	5	6.5	5	8.5	5	7.5	3.8	5.3		7.5		6.8	5.0	7.1
Timeliness	5.6	5.8	4.4	5.2	6	7	6	6	5.2	6	4.6	5.8		5.6		6	5.3	5.9
Understandability	5.5	7.5	5	6	5.8	6.5	6	9.5	7.5	7.5	6	7		6.5		7	6.0	7.2

**Figure 9:**
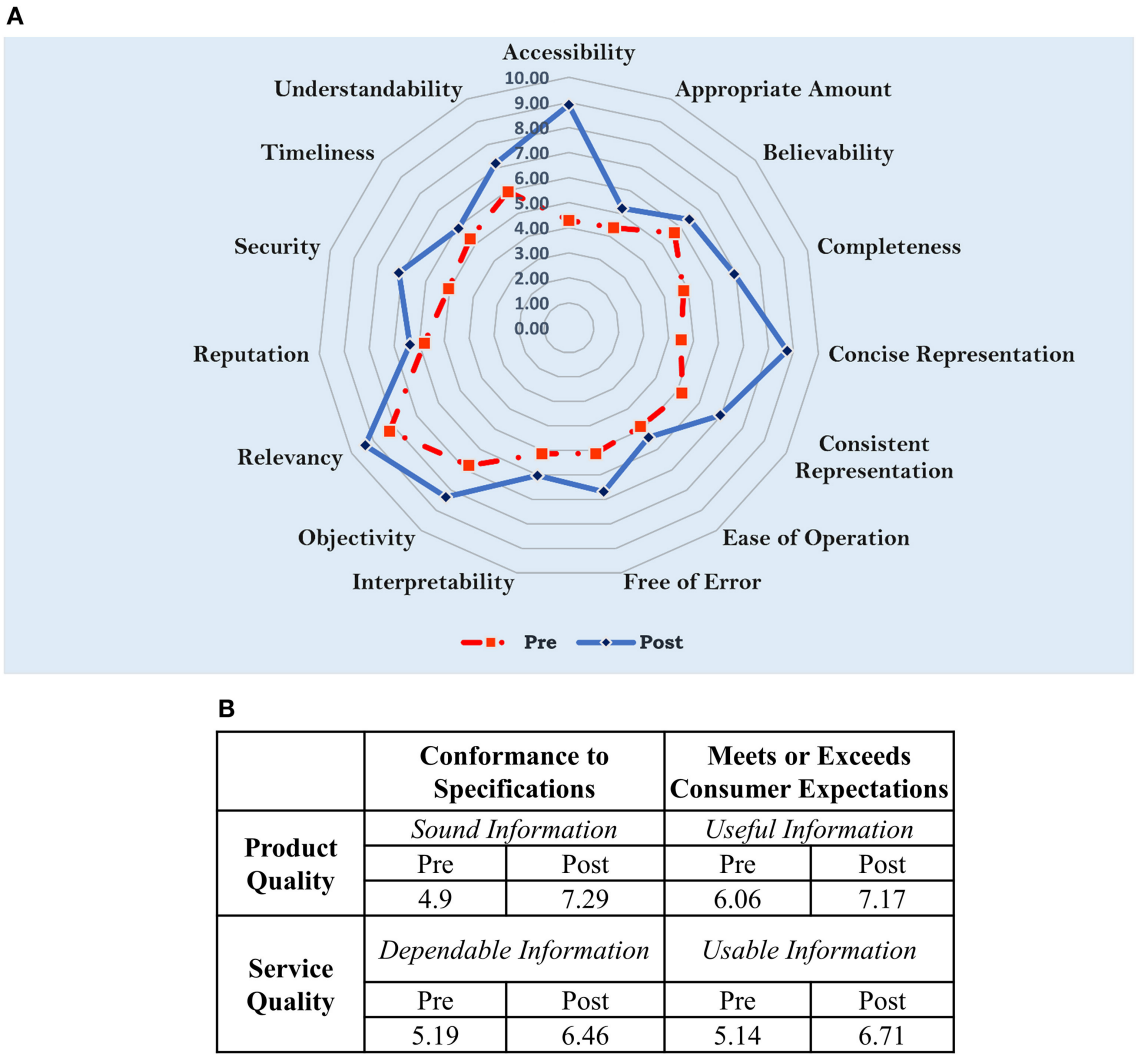
Change in pre- post-optimization information quality **(A)** impact to each information quality dimension relating to the S&C information flow **(B)** effect analyzed from the PSP/IQ model.

For further illustration, the overall impact to each information quality dimension has been visualized by the radar chart in Figure 9A and the corresponding effects to the quality of the S&C information flow analyzed from the PSP/IQ model (information as a product and service) has been illustrated by Figure 9B. The results indicate that there has been an increase in the quality of S&C information flow as a product and service.

## Discussion

This study presented results from an attempt to digitally optimize the strength and conditioning (S&C) information flow in the performance department of a professional rugby union environment. Specifically, the S&C information flow was redesigned using integral technology, activity elimination and activity automation redesign heuristics. The redesigned information flow was implemented using digital technologies (web-based applications, IoT and database management systems) by utilizing two build-measure-lean loops specified in the Lean Startup method. The internal loop was used to create minimum viable products of S&C data collection and visualization systems to meet the system functionality requirements set by their potential users. The outer loop evaluated the usability of the designed systems. For the data collection systems, usability data collected from a random sample of 50 players using the System Usability Scale (SUS) produced a mean score of 87.6 ± 10.76. And the results from statistical tests conducted for the sample against the average industry usability SUS benchmark score (68) and minimum score for a Grade A rating (80.8) for similar interfaces illustrated sufficient evidence to suggest that the data collection system could be above industry average and can be rated minimally as Grade A system. For the data visualization system, 9 cognitive walkthroughs were conducted to evaluate potential usability problems. The results helped to unravel 14 minor usability issues and 4 functionality problems. Hence, it was decided to persevere with the designed digital systems. A pre-post optimization information quality assessment of the S&C information flow conducted using the IQA questionnaire highlighted potential positive improvements in all the information quality dimensions with a distinctive enhancement to the accessibility of the S&C information flow. Similar positive improvements in the information quality were observed through the PSP/IQ model as well.

But most importantly, the case study successfully presented a practical example of how sports practitioners operating within a professional environment could effectively use existing digital technologies to optimize the information flows needed to manage professional athletes. This is critical to the current sporting landscape where the importance of managing time and cost have significantly increased due to the complexities created by the COVID-19 pandemic. Therefore, we feel that the overall outcomes from the study would be especially beneficial to practitioners requiring guidelines for implementing cost effective and easily adoptable information flow digitization solutions in sporting environments.

### Design Thinking

Previous design research clearly illustrates how technology developments fail due to poorly defined user requirements (El-Ansary, 2002; Mirza, 2010). However, for the presented study, embedding users early into the design loop when developing the digital systems allowed us to capture the expected user requirement properly and create solutions from the viewpoints of the actual end users. We believe that this was a major reason for experiencing limited challenges when deploying the designed systems into the considered professional sporting environment.

The Lean Startup framework used to design the digital systems in the current study suited the flexibility requirements of a system design expected in a dynamic environment like sports. This statement is justified since it was possible to embed changing user requirements within the internal build-measure-learn loop when developing the system MVP's (Figures 7, 8). Furthermore, the availability of high-level programming languages like R with packages like Shiny has made it easy and less time consuming to design interactive web applications like the ones developed in this study. Author JR developed all the applications by working within the considered sporting environment, this greatly assisted to bring the potential users into the design loop and allow them to be the most important member of the design team. Following a design thinking approach by modeling a system and its corresponding algorithms from standards like UML (use case, activity and sequence diagrams) helped to properly plan a system design rather than leaping straight into its implementation. Finally, we have not provided information on the data management model in the current article due to intellectual property concerns associated with sharing the relevant data architecture.

### System Usability

Evaluating the usability of a system interacting with players with time consuming methods like lab-based experiments was not practical in the considered sporting context due to the time constraints existing within the training schedule. Therefore, assessing the overall usability of a system using a quick and easy method like the System Usability Scale (SUS) was more viable in the considered environment, mainly, because it took only few minutes for a player to respond to the 10 SUS questions. Furthermore, in the current study, the use of parametric tests on Likert-scale data (like the SUS) is justified since the study adhered to the guidelines provided in literature when considering such approaches (Harpe, 2015; Mircioiu and Atkinson, 2017).

For the data visualization system, the results highlighted that most (12/14) of the usability issues identified through the cognitive walkthroughs were caused due to the poor usage of labels on the interfaces. Overall, we feel the latter usability evaluation was an important step since although sports informatics related research discusses the growing importance of data visualization in sport, less emphasis has been given to presenting results from assessments conducted to evaluate the usability of such visualization systems. Although still debated in literature, research illustrates that testing with at least 5 users could help to obtain about 80% of usability problems in a system (Nielsen et al., 1993; Nielsen, 2000). Therefore, there is a requirement to embed usability assessment of data visualization systems in professional sporting environments. In most cases, cognitive walkthroughs are normally used as a usability evaluation method without using actual users, where the design team would contextualize the interaction of the user. However, there are examples of cognitive walkthroughs conducted with actual users in research (Mahatody et al., 2010; Carvalho et al., 2014). Therefore, for the given study, since the system developments and implementation occurred within the performance department itself, it was possible to allow the actual users to perform the required tasks for the cognitive walkthroughs. But above all, by utilizing the actual users, we were able to understand the exact problems which existed in the system and improve them during deployment. So far, the deployed systems have been used for a full rugby season without the occurrence of breakdowns.

### Information Quality

The results suggested that there were improvements to all the information quality dimensions due to the optimization. However, we were unable to strictly verify the latter statement using statistical tests since the sample sizes associated with the pre-post optimization information quality assessments were low; *n* = 6 (pre) and *n* = 8 (post). Such limitations in sample size were dictated by the smaller number of HPU staff members associated with the S&C information flow. Yet, there appears to be a distinctive change in the accessibility dimension (4.3 to 8.9), suggesting a probable improvement in accessibility of player S&C data in the considered environment. The latter statement was justified by the positive feedback from the HPU staff relating to the implemented systems. Additionally, the dashboards used to monitor the implemented systems (e.g., number of connections, network usage) illustrated that the practitioners continuously used those digital systems. However, in future, there is a definite need to conduct sport informatics research for developing more objective methods for assessing information quality in sporting environments. Researchers could refer to information sciences research on objective assessments of data quality to guide such developments (Pipino et al., 2003). Finally, especially due to the optimization of the resistance information flow (Figure 5B), there were reductions in the workloads of the HPU staff (e.g., S&C Coaches).

## Conclusion

We presented the complete results from our attempts to optimize the strength and conditioning (S&C) information flow existing within the performance department of a professional rugby union environment. One of the key requirements for the optimization project considered in the study was to improve the accessibility of S&C information. Therefore, from the outcomes of the pre-post information quality assessment, we can conclude that the implemented digital systems have enhanced the S&C information flow quality with improvements to its accessibility to the HPU staff. Additionally, the build-measure-learn cycle in the Lean Startup method was a suitable framework for designing and implementing digital systems in the considered case study environment. This was due its flexibility in execution, early prototyping and short design lead times associated with the framework. In relation to the usability assessments of the systems, we can conclude that the SUS scale was a quick and efficient tool to conduct summative usability assessments of digital systems interacting with athletes in the examined professional rugby union club. Based on the findings, we can emphasize the importance of conducting evaluations for unraveling usability issues even within data visualization systems implemented to support player management. Moreover, while the IQA instrument helps to obtain a subjective assessment of information quality in a sporting environment like the one considered in this article, more objective or combined (objective and subjective) methods with less complexity and burden on the practitioners may be necessary to be developed in future due to the large increase in the implementation of digital technologies in professional sporting environments. Finally, we invite other sport researchers to utilize the methods adopted within this case study to optimize information flows within their respective environments and report the relevant findings to synthesize the outcomes that may be generalizable across different professional sporting organizations.

## Data Availability Statement

The original contributions presented in the study are included in the article/Supplementary Material, further inquiries can be directed to the corresponding author.

## Ethics Statement

The studies involving human participants were reviewed and approved by Local Research Ethics Co-ordinator, Carnegie School of Sport, Leeds Beckett University, UK. The patients/participants provided their written informed consent to participate in this study.

## Author Contributions

JR, GR, and DW contributed to conception and design of the study, read, provided revisions, and approved the submitted version. JR, MP, and MZ contributed to requirement gathering phase. JR designed all systems discussed in the study, conducted the pre- post-information quality assessment, and wrote the initial draft of the manuscript. All authors were engaged during the implementation of usability tests and evaluations.

## Conflict of Interest

JR, MZ, MP, and GR were employed by Bath Rugby Football Club. DW was employed by Leeds Rhinos Rugby League Club.

## Publisher's Note

All claims expressed in this article are solely those of the authors and do not necessarily represent those of their affiliated organizations, or those of the publisher, the editors and the reviewers. Any product that may be evaluated in this article, or claim that may be made by its manufacturer, is not guaranteed or endorsed by the publisher.
