# Supplementary material for: Digitally Optimizing the Information Flows Necessary to Manage Professional Athletes: A Case Study in Rugby Union
Source: Front Sports Act Living. 2022 Jun 9;4:850885. doi: 10.3389/fspor.2022.850885 (PMC9218428; doi:10.3389/fspor.2022.850885)
Supplement: Supplementary file 1 [file Table_1.docx]

**Supplementary Table 1.** System functionality requirements set for the initial prototype of the data visualisation interface.

| **Data Visualisation Interface** | |
| --- | --- |
| **Requirement** | **Detail** |
| Data visualisation features | A table to illustrate the all-time best scores of the entire squad (for core lifts). |
|  | A table to illustrate the current (6 weeks rolling) best scores of the entire squad (for core lifts). |
|  | Colour code current and all-time best tables according to strength standards defined at the club. |
|  | Longitudinal graphs of daily core lift data (1RM/weight/reps/heavy or dynamic) of the player for a selected date range. |
|  | Longitudinal graph illustrating if the players have done the prophylactic (yes or no) lifts at least once a week for a selected date range. |
|  | Longitudinal graphs to visualise baseline testing data of the player during a selected date range. |
|  | Colour code baseline testing data according to the club standards. |
|  | Ability to compare multiple tests on the same screen. |
|  | User authentication to access the data. |
| Database | The application must read from the database comprising of resistance training and baseline testing data. |
| User friendliness | The data visualisations must be simple and easy to use. |
| Accessibility | The interface must be easily accessible to staff during decision making. |
|  | Data colour coding standards must be accessible to staff. |
