# Supplementary material for: Digitally Optimizing the Information Flows Necessary to Manage Professional Athletes: A Case Study in Rugby Union
Source: Front Sports Act Living. 2022 Jun 9;4:850885. doi: 10.3389/fspor.2022.850885 (PMC9218428; doi:10.3389/fspor.2022.850885)
Supplement: Supplementary file 2 [file Table_2.docx]

**Supplementary Table 2.** The list of usability issues in the S&C data visualisation system unravelled from the usability evaluation.

| **No.** | **Usability problem** | **Tab** | **Improvement** | **Severity** |
| --- | --- | --- | --- | --- |
| 1 | No user feedback to notifying that data is loading after completing an action | All tabs | Add feature to show  data is loading | Minor |
| 2 | User unaware which tabs are linked to date and name inputs | All tabs | Add a method to clearly specify which tabs are linked to the name and date inputs or provide unique inputs to each tab | Minor |
| 3 | The use of 'S&C Current' tab is not clear to user | S&C Current | Change names of the tabs | Minor |
|  |  |  | Add label specifying current is 42 days rolling best scores | Minor |
| 4 | Users are confused with strength standard buttons | S&C All Time and S&C Current | Add labels to strength standard buttons | Minor |
| 5 | Colour coding legend is not obvious | S&C All Time and S&C Current | Add label to colour standard legend | Minor |
| 6 | The use of boxes in the 'S&C Charts' is unclear | S&C Charts | Add heading to the boxes specifying what they represent | Minor |
| 7 | Users struggles to find where to change the exercise type | S&C Charts | Add labels to drop down buttons | Minor |
| 8 | Uses doesn’t recognise the representation of 1RM (weight/reps) in charts | S&C Charts | Add a legend specifying 1RM = x (weight= y / reps = z) | Minor |
| 9 | Lift type (heavy, dynamic) is not recognised by user | S&C Charts | Change the label 'Lift Type' to a standard S&C name | Minor |
| 10 | User confuses with actual week number Vs season week number | S&C Charts | Change week label to standard week numbers referred by staff | Minor |
| 11 | User doesn't realise the data in the 'S&C Charts' tab | S&C Charts | Change label of 'S&C Charts' tab | Minor |
| 12 | User confused with reason for four graphs on the screen | Baseline Testing | Add label specifying four graphs can be compared | Minor |
| 13 | User does not realise what constitutes the 'Baseline Tests' tab | Baseline Testing | Change label of 'Baseline Tests' tab | Minor |
| 14 | Users are confused with baseline test standard buttons | Baseline Testing | Add labels to baseline testing standard button | Minor |

- List of identified functionality improvements.

Supplementary Table 3.2. The list of further functionality improvements requested by the users.

| **No.** | **Functionality improvement** | **Tab** | **Improvement** | **Priority** |
| --- | --- | --- | --- | --- |
| 1 | Graphical representation of 'Neck' baseline testing is suboptimal | Baseline Testing | Change graphical representation of 'Neck' baseline testing data | Low |
| 2 | Users cannot print data from the interface | All tabs | Add a button to download data as a file (e.g., csv) | Low |
| 3 | No all-time data for baseline tests | New tab | Add a tab to illustrate squad all-time best baseline testing scores, similar to 'S&C All Time' | Low |
| 4 | No daily neck strength data | S&C Charts | Collect daily neck data and visualise it | Low |
