# Supplementary material for: Digitally Optimizing the Information Flows Necessary to Manage Professional Athletes: A Case Study in Rugby Union
Source: Front Sports Act Living. 2022 Jun 9;4:850885. doi: 10.3389/fspor.2022.850885 (PMC9218428; doi:10.3389/fspor.2022.850885)
Supplement: Supplementary file 3 [file Data_Sheet_1.docx]

**Supplementary Data Sheet 1.** All statistical calculations associated with the usability assessment of the data collection system.

- SUS Questionnaire

This is a standard questionnaire that measures the overall usability of a system. Please select the answer that best expresses how you feel about each statement after using the data collection interface today.

|  | Strongly Disagree  1 | 2 | 3 | 4 | Strongly Agree  5 |
| --- | --- | --- | --- | --- | --- |
| 1. I think I would like to use this interface frequently. |  |  |  |  |  |
| 1. I found the interface unnecessarily complex. |  |  |  |  |  |
| 1. I thought the interface was easy to use. |  |  |  |  |  |
| 1. I think that I would need the support of a technical person to be able to use this interface. |  |  |  |  |  |
| 1. I found the various functions in this interface were well integrated. |  |  |  |  |  |
| 1. I thought there was too much inconsistency in this interface. |  |  |  |  |  |
| 1. I would imagine that most people would learn to use this interface very quickly. |  |  |  |  |  |
| 1. I found the interface very cumbersome to use. |  |  |  |  |  |
| 1. I felt very confident using the interface. |  |  |  |  |  |
| 1. I needed to learn a lot of things before I could get going with this interface. |  |  |  |  |  |

- The collected individual SUS scores of the 55 players.

Supplementary Table A. Collected SUS scores from the 55 players.

| **Player** | **Item** | | | | | | | | | | **Score** |
| --- | --- | --- | --- | --- | --- | --- | --- | --- | --- | --- | --- |
|  | ***Item 1*** | ***Item 2*** | ***Item 3*** | ***Item 4*** | ***Item 5*** | ***Item 6*** | ***Item 7*** | ***Item 8*** | ***Item 9*** | ***Item 10*** |  |
| ***1*** | 4 | 4 | 4 | 4 | 4 | 4 | 3 | 4 | 4 | 4 | 97.5 |
| ***2*** | 1 | 4 | 4 | 4 | 2 | 4 | 4 | 4 | 4 | 4 | 87.5 |
| ***3*** | 4 | 0 | 4 | 0 | 4 | 4 | 4 | 4 | 4 | 4 | 80 |
| ***4*** | 3 | 3 | 3 | 4 | 3 | 2 | 4 | 3 | 3 | 3 | 77.5 |
| ***5*** | 3 | 4 | 4 | 3 | 3 | 4 | 3 | 4 | 2 | 3 | 82.5 |
| ***6*** | 3 | 0 | 4 | 4 | 3 | 4 | 3 | 4 | 3 | 1 | 72.5 |
| ***7*** | 4 | 4 | 4 | 3 | 3 | 3 | 4 | 4 | 4 | 3 | 90 |
| ***8*** | 3 | 3 | 3 | 4 | 3 | 3 | 4 | 4 | 3 | 4 | 85 |
| ***9*** | 4 | 4 | 4 | 4 | 4 | 4 | 4 | 4 | 4 | 4 | 100 |
| ***10*** | 4 | 4 | 3 | 4 | 4 | 4 | 3 | 4 | 4 | 4 | 95 |
| ***11*** | 4 | 4 | 4 | 4 | 3 | 3 | 4 | 4 | 4 | 4 | 95 |
| ***12*** | 4 | 4 | 4 | 4 | 4 | 4 | 4 | 4 | 4 | 2 | 95 |
| ***13*** | 3 | 4 | 4 | 4 | 4 | 4 | 4 | 4 | 4 | 4 | 97.5 |
| ***14*** | 4 | 4 | 4 | 4 | 4 | 4 | 4 | 4 | 4 | 4 | 100 |
| ***15*** | 4 | 4 | 4 | 4 | 4 | 4 | 4 | 4 | 4 | 4 | 100 |
| ***16*** | 3 | 4 | 4 | 3 | 3 | 4 | 4 | 4 | 4 | 3 | 90 |
| ***17*** | 3 | 3 | 3 | 3 | 3 | 4 | 4 | 4 | 3 | 2 | 80 |
| ***18*** | 1 | 1 | 2 | 4 | 1 | 2 | 3 | 3 | 2 | 3 | 55 |
| ***19*** | 4 | 4 | 4 | 4 | 4 | 3 | 3 | 3 | 4 | 4 | 92.5 |
| ***20*** | 4 | 4 | 4 | 4 | 4 | 4 | 4 | 4 | 4 | 4 | 100 |
| ***21*** | 3 | 3 | 3 | 2 | 3 | 3 | 3 | 3 | 3 | 4 | 75 |
| ***22*** | 3 | 4 | 4 | 4 | 3 | 4 | 4 | 4 | 4 | 4 | 95 |
| ***23*** | 4 | 4 | 4 | 4 | 4 | 4 | 4 | 4 | 4 | 4 | 100 |
| ***24*** | 4 | 3 | 3 | 4 | 3 | 2 | 4 | 3 | 3 | 3 | 80 |
| ***25*** | 4 | 4 | 4 | 3 | 3 | 4 | 3 | 4 | 4 | 4 | 92.5 |
| ***26*** | 3 | 3 | 4 | 4 | 3 | 3 | 3 | 3 | 4 | 4 | 85 |
| ***27*** | 4 | 4 | 4 | 3 | 4 | 4 | 4 | 4 | 4 | 4 | 97.5 |
| ***28*** | 3 | 3 | 3 | 3 | 3 | 3 | 3 | 4 | 3 | 2 | 75 |
| ***29*** | 4 | 4 | 4 | 4 | 3 | 4 | 4 | 4 | 4 | 4 | 97.5 |
| ***30*** | 2 | 2 | 3 | 3 | 2 | 3 | 3 | 3 | 3 | 2 | 65 |
| ***31*** | 4 | 4 | 4 | 4 | 4 | 4 | 4 | 4 | 4 | 4 | 100 |
| ***32*** | 2 | 4 | 4 | 4 | 2 | 4 | 4 | 4 | 4 | 4 | 90 |
| ***33*** | 2 | 4 | 3 | 4 | 2 | 4 | 4 | 3 | 4 | 4 | 85 |
| ***34*** | 3 | 4 | 4 | 3 | 2 | 3 | 3 | 4 | 4 | 4 | 85 |
| ***35*** | 4 | 2 | 2 | 4 | 3 | 3 | 3 | 4 | 3 | 0 | 70 |
| ***36*** | 3 | 4 | 3 | 4 | 3 | 3 | 3 | 4 | 3 | 4 | 85 |
| ***37*** | 3 | 4 | 4 | 4 | 4 | 4 | 4 | 3 | 4 | 4 | 95 |
| ***38*** | 2 | 2 | 4 | 4 | 2 | 3 | 4 | 3 | 3 | 3 | 75 |
| ***39*** | 4 | 3 | 4 | 4 | 3 | 4 | 4 | 4 | 4 | 4 | 95 |
| ***40*** | 4 | 4 | 4 | 4 | 4 | 4 | 4 | 4 | 4 | 4 | 100 |
| ***41*** | 3 | 4 | 4 | 4 | 2 | 3 | 4 | 3 | 3 | 4 | 85 |
| ***42*** | 4 | 4 | 4 | 4 | 4 | 4 | 4 | 4 | 4 | 4 | 100 |
| ***43*** | 4 | 4 | 4 | 4 | 4 | 4 | 4 | 0 | 4 | 4 | 90 |
| ***44*** | 4 | 4 | 4 | 4 | 4 | 4 | 4 | 4 | 4 | 4 | 100 |
| ***45*** | 2 | 4 | 3 | 3 | 3 | 3 | 4 | 4 | 3 | 4 | 82.5 |
| ***46*** | 3 | 3 | 4 | 2 | 2 | 3 | 4 | 2 | 3 | 3 | 72.5 |
| ***47*** | 4 | 4 | 4 | 4 | 2 | 3 | 4 | 4 | 4 | 4 | 92.5 |
| ***48*** | 4 | 4 | 4 | 3 | 2 | 3 | 4 | 4 | 4 | 4 | 90 |
| ***49*** | 3 | 4 | 1 | 2 | 3 | 1 | 4 | 4 | 3 | 4 | 72.5 |
| ***50*** | 3 | 3 | 3 | 3 | 4 | 3 | 3 | 4 | 3 | 3 | 80 |
| ***51*** | 3 | 3 | 3 | 4 | 2 | 3 | 4 | 3 | 3 | 3 | 77.5 |
| ***52*** | 4 | 3 | 4 | 4 | 3 | 3 | 3 | 3 | 3 | 4 | 85 |
| ***53*** | 3 | 4 | 3 | 4 | 3 | 3 | 4 | 4 | 3 | 4 | 87.5 |
| ***54*** | 3 | 4 | 4 | 3 | 4 | 3 | 4 | 0 | 4 | 4 | 82.5 |
| ***55*** | 3 | 4 | 4 | 4 | 3 | 3 | 4 | 3 | 4 | 4 | 90 |

- Priori sample size calculation for at least 1 SUS score industry average score of 68.

$\alpha=0.01$ ($Z_{\frac{\alpha}{2}}=2.576)$, $SD \left( \sigma\right)=8.54 (from a similar study)$, $power=80\%$ ($Z_{\beta}$)$=0.842)$, $Population mean \left( x \right)=68, Sample mean \left( \bar{x} \right)=69$

$$sample size \left( n \right)= \frac{{(Z_{\frac{\alpha}{2}}+Z_{\beta})}^{2} \times\sigma^{2}}{{(x- \bar{x})}^{2}}$$

$$n= \frac{{(2.576+0.842)}^{2} \times{8.54}^{2}}{1^{2}}$$

$\boldsymbol{n=852}$ (not practical in any professional sporting environment)

- Normality check of the samples drawn for a population of professional rugby union players using data collection interfaces was done by drawing 1000 random samples of each size 50 from the above collected 55 SUS scores and assessing the distribution of those sample means using a histogram and Q-Q plot.


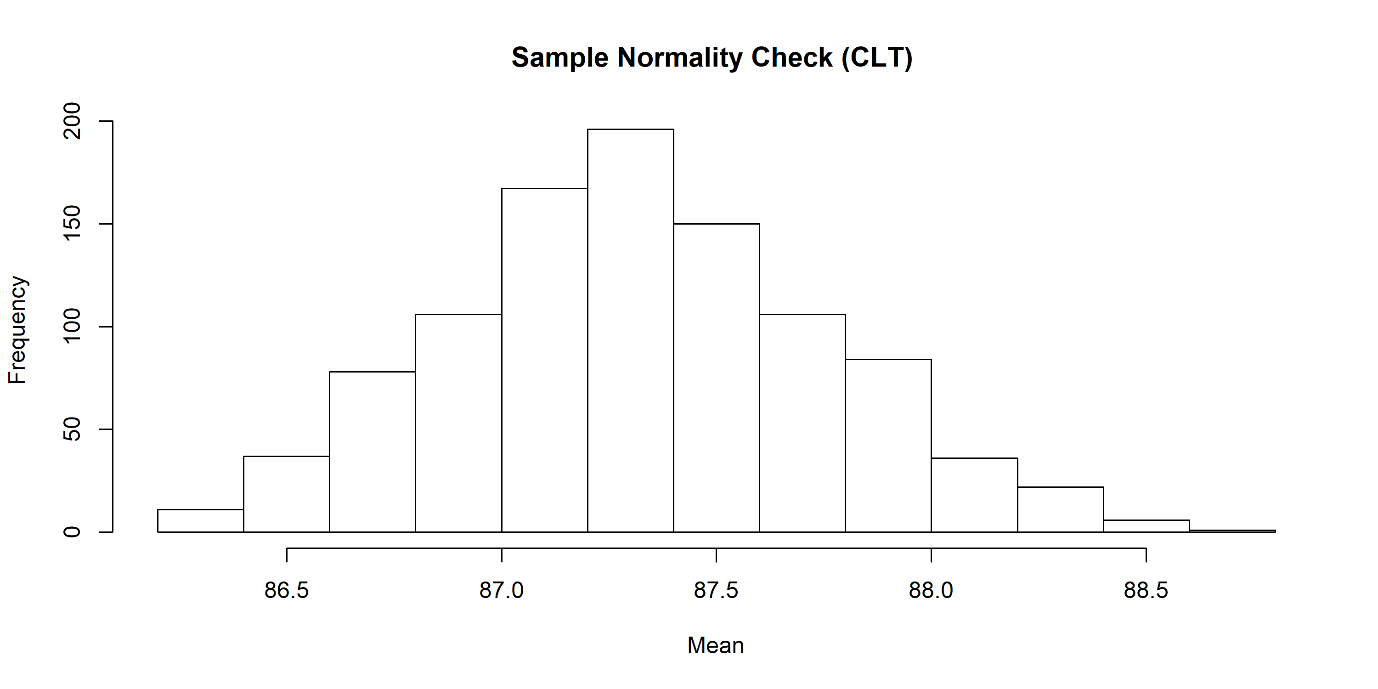


Supplementary Figure A. Histogram of the 1000 sample means.


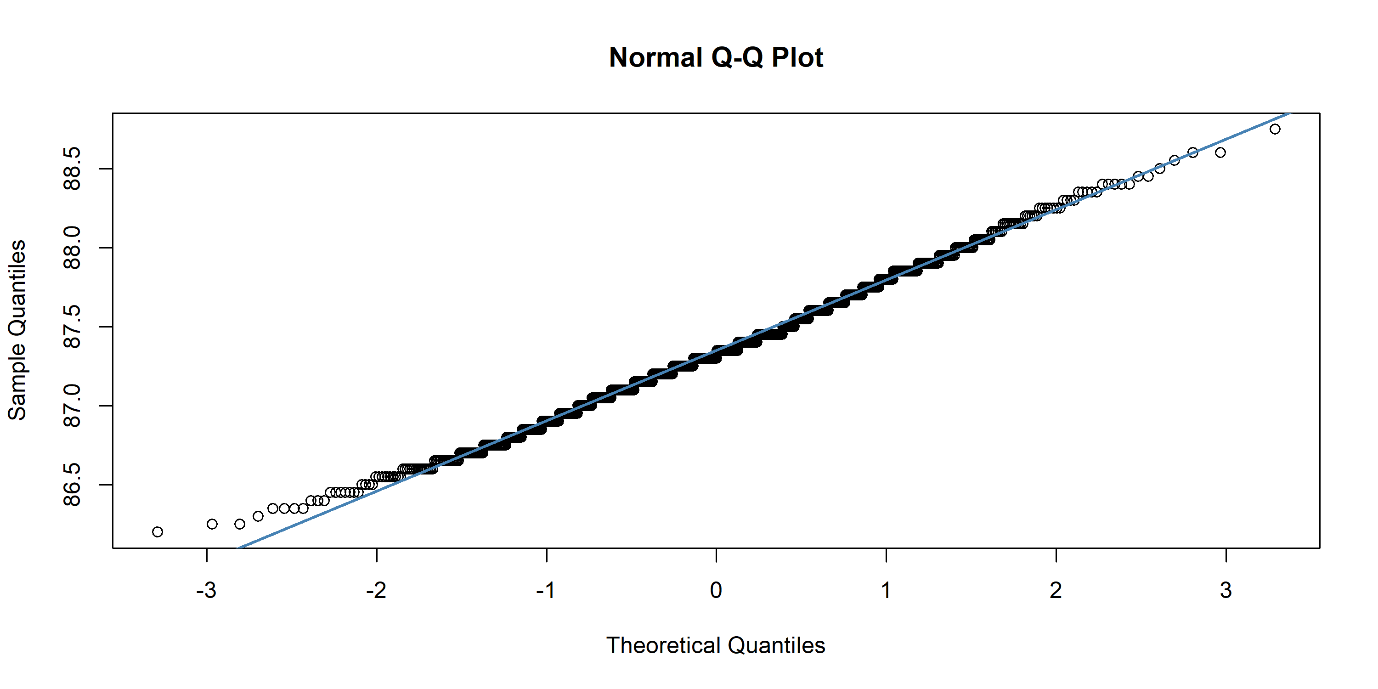


Supplementary Figure B. Sample means Q-Q plot.

- 99% Z – confidence interval (CI) of the random sample

$Mean \left( \bar{x} \right)=87.6$, $SD (\sigma)=10.76$, $confidence level= 99\%$, $n=50$

$$CI= \bar{x}\pm Z_{\frac{\alpha}{2}} \times(\frac{\sigma}{\sqrt{n}})$$

$$CI= 87.6\pm2.576 \times(\frac{10.76}{\surd50})$$

$$CI= 87.6\pm3.92$$

$$99\% CI=83.68 to 91.59$$

- Z – test to examine sample mean against average industry benchmark of 68. Similar calculation was conducted against the lower threshold (80.8) for a Grade A interface.

$Population mean \left( x \right)=68, Sample mean \left( \bar{x} \right)=87.6$, $SD (\sigma)=10.76$, $\alpha=0.01,n=50$

$$Z=\frac{\bar{x}-x}{\frac{\sigma}{\sqrt{n}}}$$

$$Z=\frac{87.6-68}{\frac{10.76}{\sqrt{50}}}$$

$$Z=12.8788$$

$$p-value=5.93 x {10}^{-38}$$

- Power calculation for the test against lower threshold (80.8) for a Grade A interface.

$Population mean \left( x \right)=68, Sample mean \left( \bar{x} \right)=87.6$, $SD (\sigma)=10.76$, $\alpha=0.005 (due to Bonferroni correction),n=50$

$$Effect size (d)=\frac{\bar{x}-x}{\sigma}$$

$$d=\frac{87.6-80.8}{10.76}$$

$$d=0.632$$

$$sample size \left( n \right)= \frac{{(Z_{\frac{\alpha}{2}}+Z_{\beta})}^{2} \times\sigma^{2}}{{(x- \bar{x})}^{2}}$$

$$Z_{\beta}=\frac{\sqrt{n}\times(x- \bar{x})}{\sigma}-Z_{\frac{\alpha}{2}}$$

$$Z_{\beta}=\frac{\sqrt{50}\times\left( 87.6-80.8 \right)}{10.76}-2.807$$

$$Z_{\beta}= 1.662$$

$$\beta= 0.0483$$

$$Power= 1- \beta=0.9517$$
