# Supplementary figures and images for: Digitally Optimizing the Information Flows Necessary to Manage Professional Athletes: A Case Study in Rugby Union
Source: Front Sports Act Living. 2022 Jun 9;4:850885. doi: 10.3389/fspor.2022.850885 (PMC9218428; doi:10.3389/fspor.2022.850885)

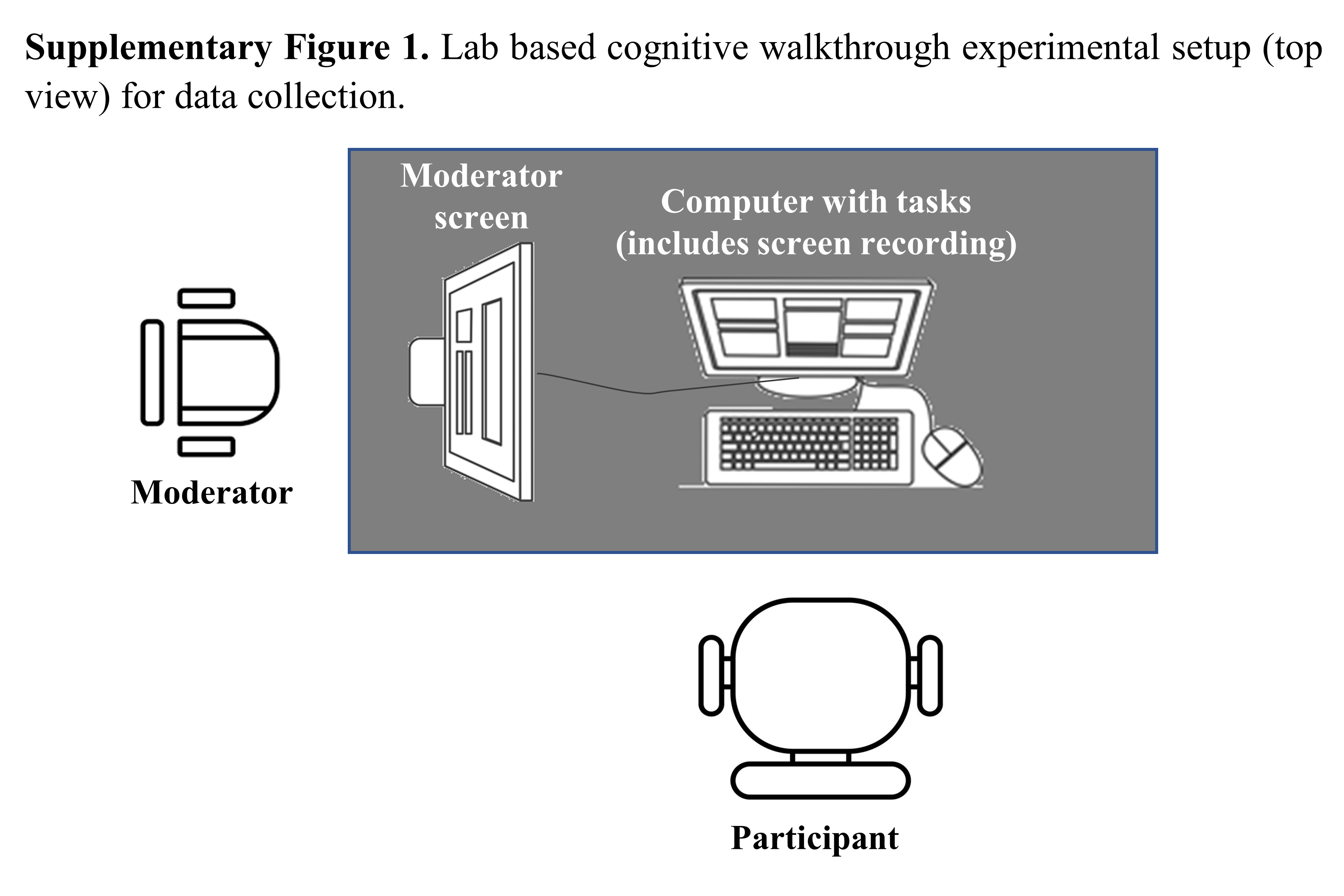

Supplement: Supplementary file 4 [file Image_1.TIF]

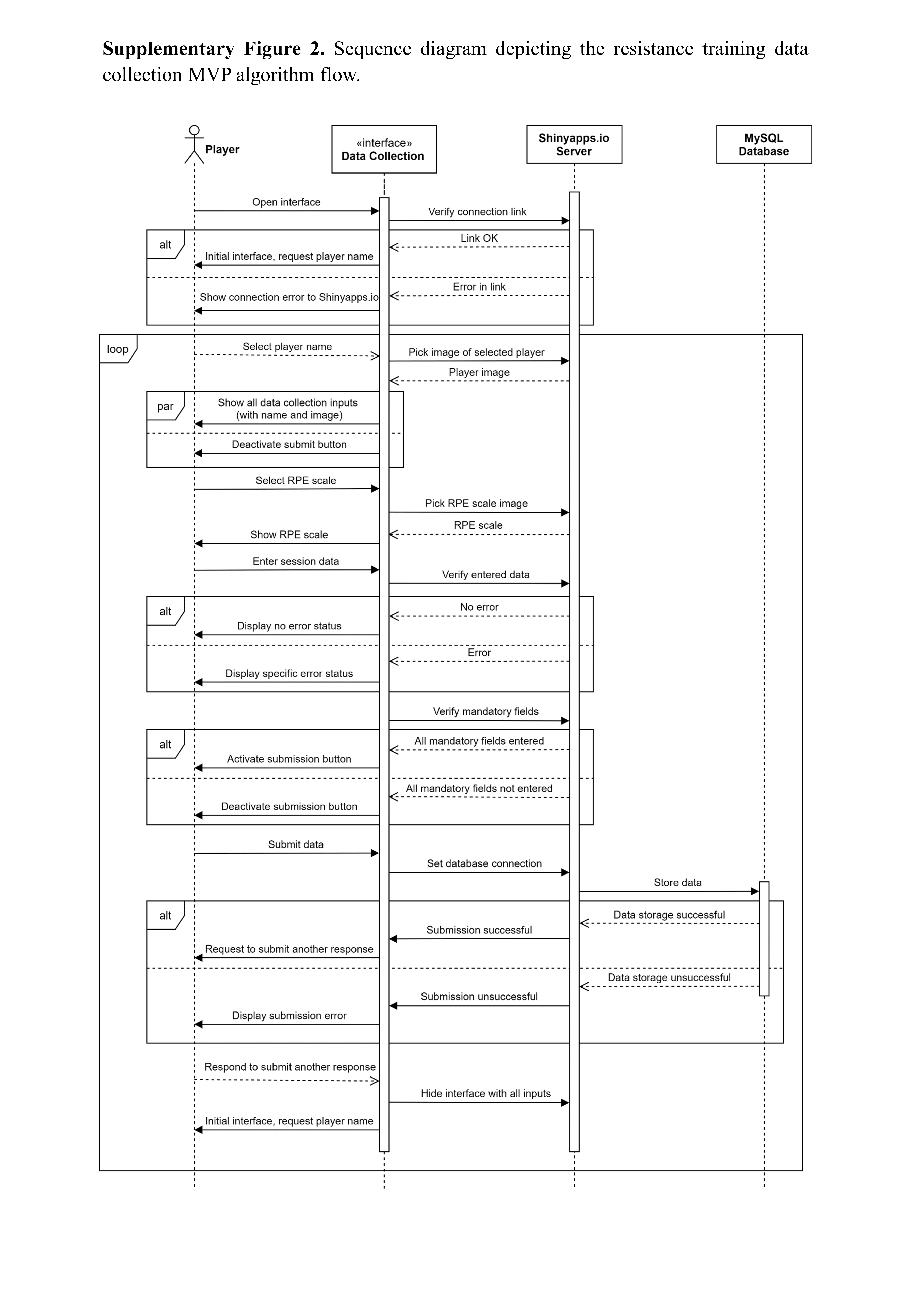

Supplement: Supplementary file 5 [file Image_2.TIF]
